# Treatment Strategy for Rifampin-Susceptible Tuberculosis

**DOI:** 10.1056/NEJMoa2212537

**Published:** 2023-02-20

**Authors:** Nicholas I. Paton, Christopher Cousins, Celina Suresh, Erlina Burhan, Ka Lip Chew, Victoria B. Dalay, Qingshu Lu, Tutik Kusmiati, Vincent M. Balanag, Shu Ling Lee, Rovina Ruslami, Yogesh Pokharkar, Irawaty Djaharuddin, Jani J.R. Sugiri, Rholine S. Veto, Christine Sekaggya-Wiltshire, Anchalee Avihingsanon, Rohit Sarin, Padmasayee Papineni, Andrew J. Nunn, Angela M. Crook, the TRUNCATE-TB Trial Team

**Affiliations:** Infectious Diseases Translational Research Programme and Yong Loo Lin School of Medicine, National University of Singapore, Singapore; London School of Hygiene and Tropical Medicine, University College London, London; Medical Research Council Clinical Trials Unit at University College London, London; Infectious Diseases Translational Research Programme and Yong Loo Lin School of Medicine, National University of Singapore, Singapore; Infectious Diseases Translational Research Programme and Yong Loo Lin School of Medicine, National University of Singapore, Singapore; Faculty of Medicine, Universitas Indonesia, and Persahabatan General Hospital, Jakarta, Indonesia; National University Hospital, Singapore; De La Salle Medical and Health Sciences Institute, Cavite, Philippines; Singapore Clinical Research Institute, Singapore; Dr. Soetomo Hospital, Surabaya, Indonesia; Lung Centre of the Philippines, Quezon City, Philippines; Singapore Clinical Research Institute, Singapore; Universitas Padjadjaran, Bandung, Indonesia; Singapore Clinical Research Institute, Singapore; Dr. Wahidin Sudirohusodo Hospital, Makassar, Indonesia; Saiful Anwar Hospital, Malang, Indonesia; Tropical Disease Foundation, Makati, Philippines; Infectious Diseases Institute, Makerere University, Kampala, Uganda; HIV-NAT, Thai Red Cross AIDS Research Center and Center of Excellence in Tuberculosis, Faculty of Medicine, Chulalongkorn University, Bangkok, Thailand; National Institute of TB and Respiratory Diseases, New Delhi, India; Infectious Diseases Translational Research Programme and Yong Loo Lin School of Medicine, National University of Singapore, Singapore; Medical Research Council Clinical Trials Unit at University College London, London; Medical Research Council Clinical Trials Unit at University College London, London

## Abstract

**Background:**

Tuberculosis is usually treated with a 6-month rifampin-based regimen. Whether a strategy involving shorter initial treatment may lead to similar outcomes is unclear.

**Methods:**

In this adaptive, open-label, noninferiority trial, we randomly assigned participants with rifampin-susceptible pulmonary tuberculosis to undergo either standard treatment (rifampin and isoniazid for 24 weeks with pyrazinamide and ethambutol for the first 8 weeks) or a strategy involving initial treatment with an 8-week regimen, extended treatment for persistent clinical disease, monitoring after treatment, and retreatment for relapse. There were four strategy groups with different initial regimens; noninferiority was assessed in the two strategy groups with complete enrollment, which had initial regimens of high-dose rifampin–linezolid and bedaquiline–linezolid (each with isoniazid, pyrazinamide, and ethambutol). The primary outcome was a composite of death, ongoing treatment, or active disease at week 96. The noninferiority margin was 12 percentage points.

**Results:**

Of the 674 participants in the intention-to-treat population, 4 (0.6%) withdrew consent or were lost to follow-up. A primary-outcome event occurred in 7 of the 181 participants (3.9%) in the standard-treatment group, as compared with 21 of the 184 participants (11.4%) in the strategy group with an initial rifampin–linezolid regimen (adjusted difference, 7.4 percentage points; 97.5% confidence interval [CI], 1.7 to 13.2; noninferiority not met) and 11 of the 189 participants (5.8%) in the strategy group with an initial bedaquiline–linezolid regimen (adjusted difference, 0.8 percentage points; 97.5% CI, −3.4 to 5.1; noninferiority met). The mean total duration of treatment was 180 days in the standard-treatment group, 106 days in the rifampin–linezolid strategy group, and 85 days in the bedaquiline–linezolid strategy group. The incidences of grade 3 or 4 adverse events and serious adverse events were similar in the three groups.

**Conclusions:**

A strategy involving initial treatment with an 8-week bedaquiline–linezolid regimen was noninferior to standard treatment for tuberculosis with respect to clinical outcomes. The strategy was associated with a shorter total duration of treatment and with no evident safety concerns. (Funded by the Singapore National Medical Research Council and others; TRUNCATE-TB ClinicalTrials.gov number, NCT03474198.)

**F**or more than four decades, the global standard treatment for drug-susceptible pulmonary tuberculosis has been a 6-month rifampin-based regimen. This treatment has cured more than 95% of persons with tuberculosis in the context of clinical trials but has underperformed in national treatment programs, in which long-term adherence is difficult for some persons and resource constraints limit the provision of adherence support.^1–3^ The unsatisfactory outcomes associated with standard treatment have contributed to the ongoing failure to meet global tuberculosis targets and to the generation of drug resistance.^4^ Exploration of new treatment approaches is essential.

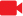

***A Quick Take is available at NEJM.org***

In clinical trials, at least 85% of participants have been cured with 3-month and 4-month regimens, and the percentage is likely to be higher when these regimens contain fluoroquinolones or rifapentine.^5–9^ A similar probability of cure has also been observed with 2-month regimens that are administered for the treatment of smear-negative tuberculosis.^5,10,11^ Thus, the current 6-month regimen may lead to overtreatment in the majority of persons in order to prevent relapse in a minority of persons. This approach may be misaligned with the desires of persons who have tuberculosis and with efficient functioning of programs, thereby impairing outcomes.

We hypothesized that a strategy involving initial treatment with an 8-week regimen, extended treatment for persistent clinical disease, follow-up after treatment, and prompt retreatment for the minority of persons who have a relapse might lead to long-term efficacy that would be noninferior to that of standard treatment, along with a reduced total duration of treatment and other secondary advantages for persons with tuberculosis and for treatment programs.

## Methods

### Trial Design and Oversight

To evaluate a treatment strategy for tuberculosis, we conducted the Two-Month Regimens Using Novel Combinations to Augment Treatment Effectiveness for Drug-Sensitive Tuberculosis (TRUNCATE-TB) trial, a seamless phase 2–3, prospective, multicenter, international, adaptive, multigroup, multistage, randomized, open-label, noninferiority trial with a 96-week follow-up period. Because it was a strategy-comparison trial, the design and the approach to analysis differed from those used in regimen-comparison trials. The trial was designed by the investigators and coordinated by investigators at the National University of Singapore. Sanofi donated rifapentine, Pfizer donated linezolid, and Janssen funded whole-genome sequencing; these companies had no role in the design or conduct of the trial. An independent trial steering committee provided oversight. An independent data and safety monitoring committee reviewed safety and interim efficacy data. National and local ethics committees and regulatory agencies approved the trial. All participants provided written informed consent. The authors vouch for the accuracy and completeness of the data and the fidelity of the trial to the protocol, available with the full text of this article at NEJM.org.

### Trial Population

Persons were eligible for inclusion in the trial if they were 18 to 65 years of age, had symptoms of tuberculosis or evidence of tuberculosis on a chest radiograph, and had a nucleic acid amplification test (Xpert MTB/RIF test, Cepheid) that was positive for tuberculosis without rifampin resistance. Persons who had a grade 3+ sputum smear, a cavity measuring more than 4 cm on a chest radiograph, or a positive test for human immunodeficiency virus (HIV) antibodies were initially not eligible; these exclusion criteria were later removed. A complete list of eligibility criteria and details regarding the changes are provided in Section S1 in the Supplementary Appendix, available at NEJM.org.

### Randomization and Treatment Strategy

Participants were randomly assigned to undergo either standard treatment or a strategy involving initial treatment with an 8-week regimen, extended treatment for persistent clinical disease, monitoring after treatment, and retreatment for relapse. There were four strategy groups with different initial regimens; participants were randomly assigned to the standard-treatment group or to one of the four strategy groups in equal proportions. Randomization was conducted by site staff with the use of an online system and was stratified according to trial site and relapse risk (Section S2).

Standard treatment consisted of a standard dose of rifampin and isoniazid for 24 weeks in combination with pyrazinamide and ethambutol for the first 8 weeks. In the four strategy groups, initial treatment consisted of the following 8-week regimens: a high dose of rifampin and linezolid, a high dose of rifampin and clofazimine, rifapentine and linezolid, and bedaquiline and linezolid, each in combination with isoniazid, pyrazinamide, and ethambutol. In the strategy group with an initial rifapentine–linezolid regimen, ethambutol was replaced with levofloxacin (Section S3). The rationale for regimen selection is described in the protocol. The high dose of rifampin was 35 mg per kilogram of body weight initially and was reduced to 20 mg per kilogram starting on November 1, 2019. When a participant had persistent clinical disease (symptoms and a positive sputum smear) at week 8 or had missed doses, treatment with the five-drug regimen could be extended through week 12. When a participant had persistent clinical disease at week 12 or had adverse events at an earlier point, the five-drug regimen could be switched to standard treatment to complete a 24-week course of treatment.

Treatment was supervised on a daily basis at least until completion of the four-drug phase in the standard-treatment group or until completion of the five-drug regimen in the four strategy groups. The approach to supervision was tailored to the participant.

Monitoring involved assessment for symptoms and examination of sputum smears. Results of sputum cultures were also provided to clinicians, and additional tests were performed for suspected relapse. Participants who met prespecified criteria for relapse (Section S4) were retreated for at least 24 weeks with standard treatment, which was adjusted according to the participant’s resistance profile.

The trial design anticipated discontinuation of enrollment in two strategy groups on the basis of early stopping rules. However, the data and safety monitoring committee did not recommend discontinuation of enrollment in any trial group at the time of the interim analyses. The trial steering committee discontinued enrollment in two strategy groups (the rifampin–clofazimine strategy group and the rifapentine–linezolid strategy group) to ensure that sample-size requirements could be met for the formal evaluation of noninferiority in the two remaining strategy groups. The selection of these groups was pragmatic, with blinding to outcome data; the decision was based on pill burden, regulatory advice, and import license restrictions (Section S5).

### Assessments and Outcomes

Clinic visits were scheduled every 1 to 4 weeks through week 24, then every 12 weeks through week 96; starting at week 30, monthly telephone visits were interspersed between clinic visits (Section S6). At every visit, tuberculosis symptoms were reviewed with a standard checklist, adverse events were graded according to standard criteria,^12^ and adherence to treatment was assessed on the basis of treatment records and participant interviews. A chest radiograph was obtained at screening, at weeks 8 and 96, at the end of treatment, and when relapse was suspected. Respiratory disability was assessed at week 96 with the use of the Medical Research Council (MRC) breathlessness scale, with disability defined as a grade of 3 or higher (on a scale from grade 1 to grade 5, with higher grades indicating a greater degree of activity-related breathlessness), and by means of spirometry, with disability defined as a forced expiratory volume in 1 second (FEV_1_) of less than 50% of the predicted value (Section S7).

Sputum was obtained for smear examination and liquid culture (Mycobacteria Growth Indicator Tube system, Becton Dickinson) at every visit and when relapse was suspected. Smears were examined according to the method in routine use at each trial site and were graded according to World Health Organization guidelines. Drug resistance was determined with phenotypic susceptibility testing for standard drugs at baseline and for drugs associated with previous exposure at relapse (Section S8). Whole-genome sequencing was performed on isolates obtained at baseline and at relapse. Finally, acceptability was assessed with a questionnaire at weeks 48 and 96 (Section S9).

The primary outcome was a composite of death before week 96 or ongoing tuberculosis treatment or active tuberculosis at week 96. The primary outcome was assessed with a prespecified algorithm (Section S10). Because detection of and retreatment for relapse are an integral part of the treatment strategy that was assessed in this trial, these outcomes were not considered to be primary-outcome events if retreatment had been completed and the participant did not have active disease at week 96. Secondary outcomes included participant-centered, safety, and program-centered outcomes. The main secondary outcomes were total treatment time, grade 3 or 4 adverse events, and acquired drug resistance. Details regarding the conduct of the trial are provided in the protocol, which includes the statistical analysis plan.

### Statistical Analysis

We estimated that a sample of 180 participants in each trial group with complete enrollment (i.e., groups in which enrollment was not discontinued before the sample-size requirement was met) would provide the trial with 85% power to show the noninferiority of the treatment strategy to standard treatment with respect to the risk of a composite of death, ongoing treatment, or active disease at week 96. This estimation was based on a noninferiority margin of 12 percentage points, a one-sided significance level of 0.0125 (an adjustment for multiplicity, with the assumption of complete enrollment in two strategy groups), and the exclusion of 10% of participants from the analysis population. We assumed that a primary-outcome event would occur in 10% of the participants in each trial group (Section S11).

All analyses were performed in the intention-to-treat population, which excluded only persons who underwent randomization in error and were withdrawn before any trial medication was administered. Formal hypothesis testing was performed in only the two strategy groups with complete enrollment. Noninferiority of a treatment strategy could be concluded if the upper limit of the two-sided 97.5% confidence interval for the difference between the strategy group and the standard-treatment group in the percentage of participants with a primary-outcome event was less than 12 percentage points (Section S11). Differences were estimated with a generalized linear model with binomial distribution and adjustment for country and relapse risk.

Analysis of the primary outcome was performed across multiple subgroups that were defined according to baseline characteristics. Pre-specified sensitivity analyses were performed in the assessable population, which excluded participants who had an outcome that was classified as unassessable, and in the per-protocol population, which excluded participants who did not complete the protocol-specified initial treatment or had inadequate treatment during the first 56 days, unless the reason for inadequate treatment was death. The widths of confidence intervals that are used to report secondary outcomes have not been adjusted for multiplicity, and the intervals may not be used in place of hypothesis testing. Details regarding the reporting of primary and secondary outcomes and the approach to missing data are provided in Sections S12 and S13 and the statistical analysis plan. All analyses were performed with SAS software, version 9.4 (SAS Institute).

## Results

### Participants

**Figure 1:**
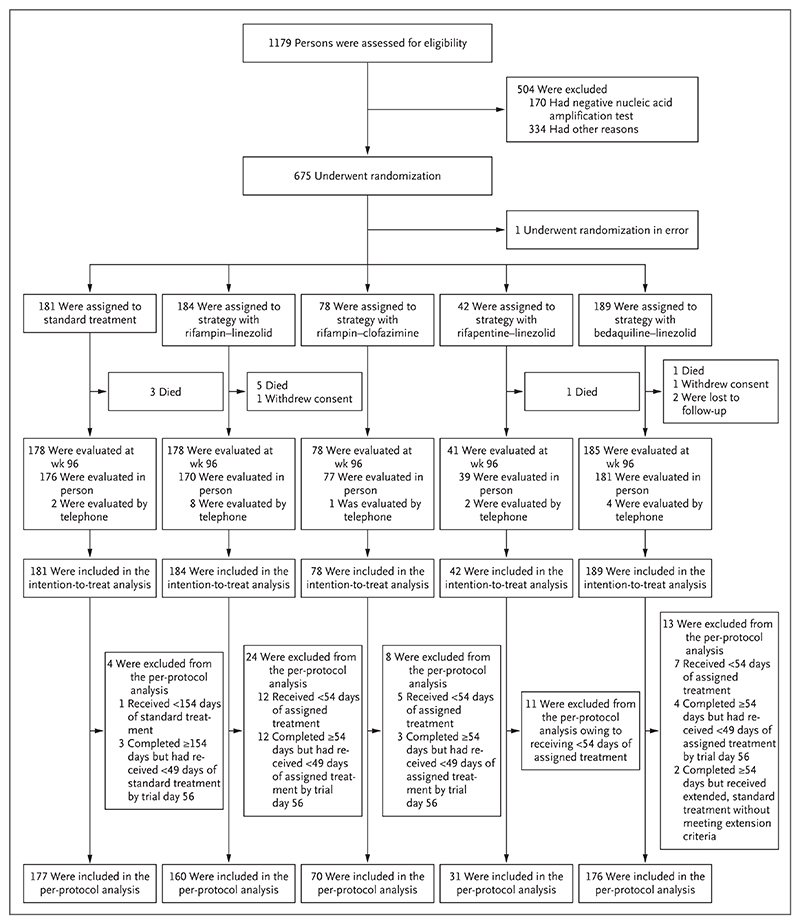
Screening, Randomization, Evaluation, and Analysis. In the standard-treatment group, two participants received less than 154 days of treatment because they died; these participants were not excluded from the per-protocol analysis.

From March 21, 2018, through January 20, 2020, a total of 1179 participants were screened and 675 were enrolled at 18 sites in Indonesia, the Philippines, Thailand, Uganda, and India. One person underwent randomization in error and was withdrawn immediately. Of the 674 participants who were included in the intention-to-treat population, 4 (0.6%) withdrew consent or were lost to follow-up (Fig. 1). The characteristics of the participants at baseline were similar in all five trial groups (Table 1), and the groups were broadly representative of populations of persons with tuberculosis (Table S1). All 660 participants who were alive and undergoing follow-up were evaluated at week 96; of these, 643 (97.4%) were evaluated in person and 17 (2.6%) by telephone.

In the standard-treatment group, 98.3% of the participants completed the 24-week treatment course, and 3.3% underwent retreatment (Tables S2 and S3). In the four strategy groups, 91.5% of the participants overall (range, 73.8 to 94.7) completed the initial 8-week treatment course and stopped (mean qualifying time of initial treatment, 58 days), 6.5% overall switched to standard treatment (mainly because of adverse events) and completed a 24-week course, and 17.0% overall (range, 12.7 to 22.8) underwent retreatment.

### Primary Efficacy Outcome

**Figure 2:**
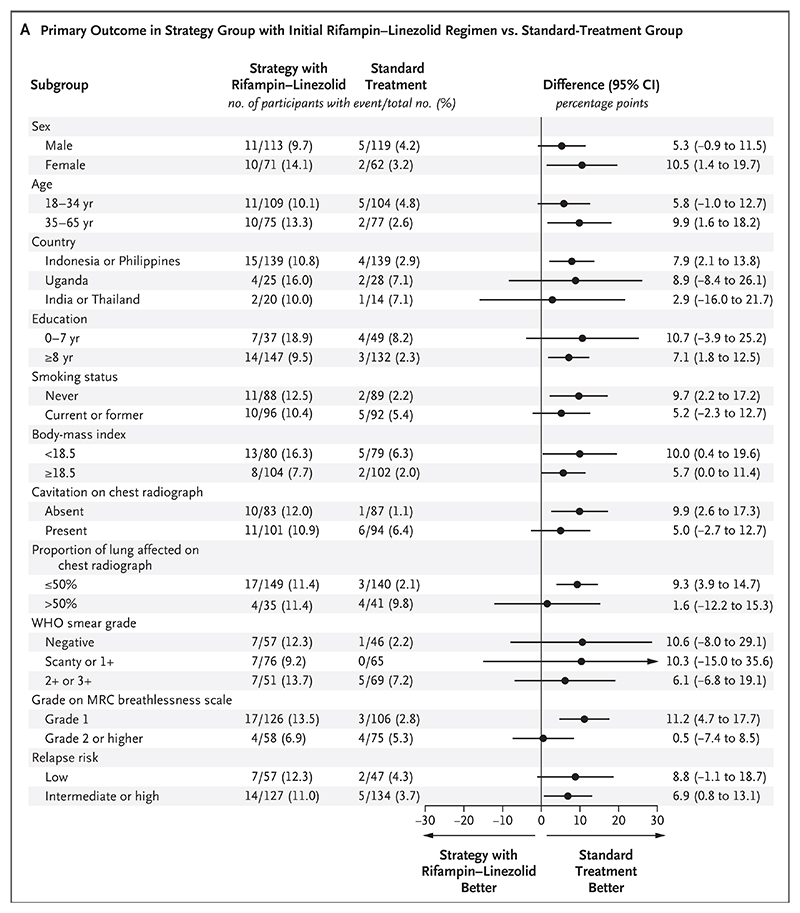

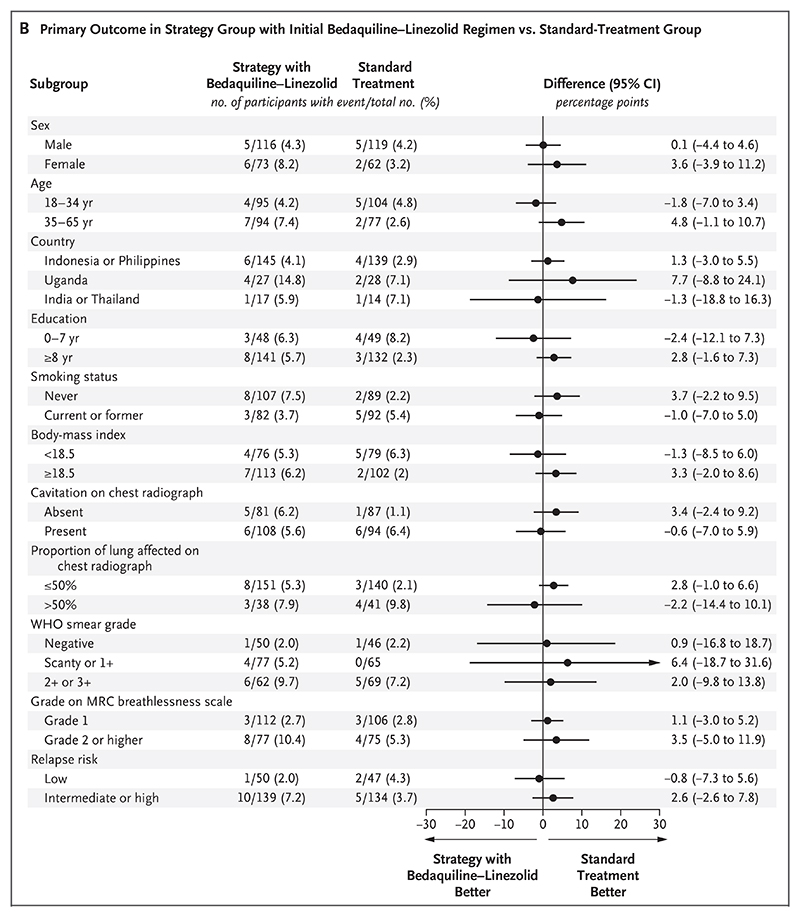
Subgroup Analysis. Shown is the percentage of participants who had a primary-outcome event in the strategy group with an initial rifampin–linezolid regimen (Panel A) and in the strategy group with an initial bedaquiline–linezolid regimen (Panel B), as compared with the standard-treatment group, according to prespecified subgroups. Differences were estimated with a generalized linear model with adjustment for country. The widths of the confidence intervals have not been adjusted for multiple comparisons, and the intervals cannot be used to infer treatment effects. In a post hoc subgroup analysis, the estimated difference between the rifampin–linezolid strategy group and the standard-treatment group in the percentage of participants with a primary-outcome event was 4.6 percentage points (95% CI, −2.7 to 12.0) among those who were enrolled before the high dose of rifampin was reduced and 10.6 percentage points (95% CI, 3.2 to 18.1) among those who were enrolled after the rifampin dose reduction. Body-mass index is the weight in kilograms divided by the square of the height in meters. The Medical Research Council (MRC) breathlessness scale ranges from grade 1 to grade 5, with higher grades indicating a greater degree of activity-related breathlessness. For relapse risk, low risk is defined as a negative smear and the absence of a cavity measuring more than 4 cm on a chest radiograph; intermediate risk as a positive smear of grade 2+ or lower and the absence of a cavity measuring more than 4 cm on a chest radiograph; and high risk as a positive smear of grade 3+, the presence of a cavity measuring more than 4 cm on a chest radiograph, or both. WHO denotes World Health Organization.

In the intention-to-treat analysis, a primary-out-come event occurred in 7 of the 181 participants (3.9%) in the standard-treatment group, as compared with 21 of the 184 participants (11.4%) in the strategy group with an initial rifampin–linezolid regimen (adjusted difference, 7.4 percentage points; 97.5% confidence interval [CI], 1.7 to 13.2; noninferiority criterion not met) and 11 of the 189 participants (5.8%) in the strategy group with an initial bedaquiline–linezolid regimen (adjusted difference, 0.8 percentage points; 97.5% CI, −3.4 to 5.1; noninferiority criterion met) (Table 2). Sensitivity analyses that were performed in the assessable population and in the per-protocol population had similar results. The results were also consistent across prespecified subgroups, including those defined according to relapse risk (Fig. 2A and 2B). The estimated percentages of participants who had a primary-outcome event in the two strategy groups with incomplete enrollment (10.3% and 4.8% in the intention-to-treat analysis) were similar to the percentages observed in the two strategy groups with complete enrollment. Results for the primary efficacy outcome are shown in Tables S4 through S9.

### Participant-Centered Secondary Outcomes

The mean total duration of treatment through week 96 was 180 days in the standard-treatment group, 106 days in the strategy group with an initial rifampin–linezolid regimen, and 85 days in the strategy group with an initial bedaquiline–linezolid regimen (Table 3). Participants in the strategy groups reported low levels of difficulty and anxiety related to the strategy and reported that the strategy had a positive effect on their motivation to take treatment; most participants in the strategy groups (71.6% and 78.3%) indicated that they would recommend the strategy to others. For other participant-centered outcomes, the results in the two strategy groups with complete enrollment were similar to those in the standard-treatment group. The results for participant-centered outcomes in the two strategy groups with incomplete enrollment were similar to those in the other two strategy groups. Results for participant-centered outcomes are shown in Table S10 and Fig. S1.

### Safety Outcomes

The incidences of grade 3 or 4 adverse events, serious adverse events, and death did not differ significantly between the standard-treatment group and the two strategy groups with complete enrollment (Table 3). The incidence of respiratory disability and the change in the FEV_1_ through week 96 were also similar in the three groups. In the strategy group with an initial rifampin–linezolid regimen, the incidence of grade 3 or 4 adverse events was similar among participants enrolled before the high dose of rifampin was reduced and those enrolled after the rifampin dose reduction. Almost all relapses were classified as grade 1 or 2 in severity; 1 participant in the standard-treatment group, 5 in the rifampin–linezolid strategy group, and 1 in the bedaquiline–linezolid strategy group had a grade 3 or 4 adverse event that was related to a relapse or retreatment episode. The results for safety outcomes in the two strategy groups with incomplete enrollment were similar to those in the other two strategy groups. Results for safety outcomes are shown in Tables S11 through S15.

### Program-Centered Secondary Outcomes

Within the first 56 days, the mean percentage of days on which participants took the prescribed drugs was at least 95% in each trial group (Table 3). Few participants had treatment cessation that started during the first 56 days and lasted for at least 56 consecutive days; such cessation of treatment occurred at a similar frequency across trial groups. Two participants, both of whom were in the strategy group with an initial bedaquiline–linezolid regimen, had confirmed acquired drug resistance. One of these participants had resistance to isoniazid at baseline, missed 14 days (including 12 consecutive days) of treatment with all drugs during the first 4 weeks, and had a relapse at week 52. The other completed treatment at week 8 with no missed doses and had a relapse at week 36. Both had phenotypic and genotypic resistance to bedaquiline, as well as to clofazimine. Retreatment with standard treatment (with levofloxacin added for the first participant) was successful. Cases of unconfirmed acquired resistance to pyrazinamide and isoniazid are listed in Table S16. The estimated risk of relapse-associated transmission was low in the two strategy groups, with a mean transmission risk period of 3 days and with potential exposure of less than 0.1 additional new household contact per participant during the 96 weeks of follow-up. The results for program-centered outcomes in the two strategy groups with incomplete enrollment were similar to those in the other two strategy groups. Results for program-centered outcomes are shown in Table S11.

## Discussion

The results of the TRUNCATE-TB trial showed that a strategy involving initial treatment with an 8-week regimen that contained bedaquiline and linezolid was noninferior to standard treatment with respect to the risk of a composite clinical outcome at week 96. The efficacy of the strategy, as compared with standard treatment, was consistent across multiple subgroups that were defined according to baseline characteristics, including some that are indicative of severe disease and high relapse risk.

This treatment strategy was associated with a shorter initial course and with a shorter total duration of treatment than was standard treatment. Also, participants who were treated according to this strategy reported a higher level of motivation to adhere to an 8-week initial course than to standard treatment. The 13-week reduction in the total treatment duration could allow program resources (both financial and human) — which are currently consumed by procuring, distributing, and supervising additional months of treatment — to be redeployed to enhance adherence support during a shorter period. This support may synergize with the increased individual motivation to better sustain adherence and thereby prevent the decrease in effectiveness that has been seen with standard treatment in its translation from clinical trials to programs.

Follow-up after treatment, which is an essential component of the strategy, represents an additional burden for persons with tuberculosis and for treatment programs, as compared with the usual practice of immediate discharge after completion of standard treatment. However, only a few participants discontinued visits or reported difficulty with prolonged follow-up; most indicated that they would recommend the strategy to others, which suggests a positive overall experience, and the pragmatic monitoring approach is likely to be feasible for treatment programs. Future cost-effectiveness analyses to explore whether the additional costs of monitoring after treatment and associated retreatment are offset by the costs saved with reduced treatment duration are under way.

Our finding of no substantive overall increase in the incidence of grade 3 or 4 adverse events, serious adverse events, or respiratory disability (which has been previously observed with recurrent tuberculosis^13^) supports the premise that follow-up after treatment and early detection of recurrence mitigate the risk of harm from excess relapses. Overall, there was no evidence that the strategy promoted drug resistance, although the trial sample size was small; the finding of infrequent drug resistance was consistent with findings in previous trials of 4-month rifamycinbased regimens, in which less than 1% of participants acquired rifamycin resistance.^9,14^ A particular theoretical concern was that the long half-lives of bedaquiline and clofazimine (which are several months^15,16^) might result in exposure of residual viable bacteria to monotherapy after a short treatment course and generate frequent drug resistance. However, only two participants (1.1%) acquired bedaquiline (and clofazimine) resistance; this incidence is lower than the 2% incidence reported with bedaquiline use in 6-month regimens.^17,18^ One of these participants missed multiple consecutive treatment doses early in the course of treatment, which is a risk regardless of the regimen.^19,20^ The additional risk of transmission of tuberculosis with the strategy also appears to be small, with mitigation by follow-up after treatment and by early detection and treatment of relapses.

This treatment strategy could be refined with the use of alternative drug regimens or monitoring approaches. Any initial regimen that has an acceptable side-effect profile and constrains relapse at modest levels may be suitable. Of the two strategy groups that were formally evaluated for noninferiority, only the group that was assigned to receive initial treatment with a bedaquiline–linezolid regimen met the noninferiority criterion at week 96. Bedaquiline may be well-suited for a 2-month initial course because its long half-life may extend efficacy beyond treatment completion, with a low risk of drug resistance. We used bedaquiline with four companion drugs to maximize potency and minimize the risk of resistance. We used a dose and duration of linezolid that were similar to those associated with safety among persons with drug-resistant tuberculosis,^21^ and the course was completed by more than 95% of participants who received it. Future analyses to evaluate the safety and efficacy of all the regimens used in this trial with respect to conventional phase 2 and 3 outcomes and pharmacokinetic–pharmacodynamic models are under way.

For monitoring, we used regular assessment of symptoms and examination of sputum smears to inform decisions regarding treatment extension and to identify participants who should undergo additional investigation for relapse, which is a pragmatic approach that would be feasible for programs. We also provided clinicians with the results of cultures, which were performed primarily for research purposes. Future analyses might examine the extent to which culture results influence clinical management and whether other biomarkers might simplify, improve, or accelerate decision making.^22,23^ A point-of-care, non–sputum-dependent biomarker of disease activity (such as a blood RNA signature^24,25^) could work well with this strategy.

The main strengths of this trial are the pragmatic design, the use of outcome measures that are relevant to persons with tuberculosis and to treatment programs, and the inclusion of diverse treatment clinics in high-burden countries, mainly in Asia. The open-label design is a limitation, but it was the most feasible option for regimens of different durations. The use of standardized assessments, the use of a prespecified algorithm for primary-outcome assessment, and the negligible trial attrition minimize potential bias. No HIV-positive participants were enrolled (although enrollment of such participants was permitted later in the trial), and further evaluation in this population is warranted.

The results of this trial suggest that there may be value in considering a shift in tuberculosis management to a strategy involving initial treatment for the minimum duration needed to cure the majority of persons with tuberculosis, extended treatment for persistent clinical disease, and monitoring after treatment to detect relapse in the minority of persons who need retreatment. This treatment strategy provides a framework for the development of new, short, potent drug regimens and biomarkers for treatment monitoring to maximize cost-effectiveness and outcome benefit. Implementation research is vital to refine the strategy and evaluate outcomes in individual treatment programs and diverse populations before consideration of adoption at scale.

## Supplementary Material

Supplementary appendix

## Figures and Tables

**Table 1:** Baseline Characteristics of the Participants in the Intention-to-Treat Population.*

Characteristic	Standard Treatment (N = 181)	Strategy with Rifampin– Linezolid (N = 184)	Strategy with Rifampin– Clofazimine (N = 78)†	Strategy with Rifapentine– Linezolid (N = 42)†	Strategy with Bedaquiline– Linezolid (N = 189)	Overall (N = 674)
Male sex — no. (%)	119 (66)	113 (61)	48 (62)	25 (60)	116 (61)	421 (62)
Age group — no. (%)						
18–34 yr	104 (57)	109 (59)	51 (65)	26 (62)	95 (50)	385 (57)
35–50 yr	59 (33)	57 (31)	21 (27)	11 (26)	70 (37)	218 (32)
51–65 yr	18 (10)	18 (10)	6 (8)	5 (12)	24 (13)	71 (11)
Country — no. (%)						
Indonesia	78 (43)	73 (40)	38 (49)	23 (55)	82 (43)	294 (44)
Philippines	61 (34)	66 (36)	32 (41)	15 (36)	63 (33)	237 (35)
Thailand	10 (6)	15 (8)	8 (10)	4 (10)	12 (6)	49 (7)
Uganda	28 (15)	25 (14)	0	0	27 (14)	80 (12)
India	4 (2)	5 (3)	0	0	5 (3)	14 (2)
Median body weight (range) — kg	50 (32–81)	50 (30–97)	48 (35–88)	50 (32–71)	50 (32–86)	50 (30–97)
Median body-mass index (range)‡	19 (14–29)	19 (14–33)	19 (14–29)	18 (12–25)	19 (13–30)	19 (12–33)
Body-mass index — no. (%)‡						
<17	39 (22)	42 (23)	21 (27)	13 (31)	47 (25)	162 (24)
17 to <18.5	40 (22)	38 (21)	14 (18)	9 (21)	29 (15)	130 (19)
≥18.5	102 (56)	104 (57)	43 (55)	20 (48)	113 (60)	382 (57)
Employment status — no. (%)						
Working full or part time	94 (52)	99 (54)	35 (45)	16 (38)	100 (53)	344 (51)
Student	10 (6)	15 (8)	10 (13)	10 (24)	15 (8)	60 (9)
Not working	77 (43)	70 (38)	33 (42)	16 (38)	74 (39)	270 (40)
Current smoker — no. (%)	34 (19)	33 (18)	15 (19)	8 (19)	31 (16)	121 (18)
Former smoker — no. (%)	58 (32)	63 (34)	24 (31)	13 (31)	51 (27)	209 (31)
Proportion of lung affected on chest radiograph — no. (%)						
<25%	46 (25)	62 (34)	28 (36)	12 (29)	53 (28)	201 (30)
25–50%	94 (52)	87 (47)	36 (46)	24 (57)	98 (52)	339 (50)
>50%	41 (23)	35 (19)	14 (18)	6 (14)	38 (20)	134 (20)
Cavitation on chest radiograph — no. (%)						
Absent	87 (48)	83 (45)	41 (53)	19 (45)	81 (43)	311 (46)
Largest cavity ≤4 cm	90 (50)	96 (52)	37 (47)	23 (55)	106 (56)	352 (52)
Largest cavity >4 cm	4 (2)	5 (3)	0	0	2 (1)	11 (2)
WHO smear grade — no./total no. (%) §						
Negative	46/180 (26)	57/184 (31)	26/78 (33)	12/41 (29)	50/189 (26)	191/672 (28)
Scanty	27/180 (15)	28/184 (15)	12/78 (15)	7/41 (17)	24/189 (13)	98/672 (15)
1+	38/180 (21)	48/184 (26)	25/78 (32)	13/41 (32)	53/189 (28)	177/672 (26)
2+	44/180 (24)	37/184 (20)	8/78 (10)	7/41 (17)	38/189 (20)	134/672 (20)
3+	25/180 (14)	14/184 (8)	7 /78 (9)	2/41 (5)	24/189 (13)	72/672 (11)
Bacillary burden on nucleic acid amplification test — no./total no. (%)¶						
Very low	25/173 (14)	22/172 (13)	8/74 (11)	3/37 (8)	16/184 (9)	74/642 (12)
Low	40/173 (23)	48/172 (28)	22/74 (30)	11/37 (30)	52/184 (28)	173/642 (27)
Medium	72/173 (42)	80/172 (47)	31/74 (42)	15/37 (41)	73/184 (40)	271/642 (42)
High	36/173 (21)	22/172 (13)	13/74 (18)	8/37 (22)	43/184 (23)	122/642 (19)
Positive sputum culture — no. (%)	166 (92)	168 (91)	68 (87)	39 (93)	171 (90)	612 (91)
Drug resistance — no./total no. (%)‖						
Isoniazid	12/162 (7)	15/166 (9)	5/68 (7)	2/39 (5)	12/169 (7)	46/604 (8)
Pyrazinamide	5/133 (4)	2/135 (1)	5/54 (9)	1/29 (3)	5/136 (4)	18/487 (4)
Ethambutol	1/162 (1)	0	2/68 (3)	0	2/169 (1)	5/604 (1)
Relapse risk — no. (%)**						
Low	47 (26)	57 (31)	26 (33)	13 (31)	50 (26)	193 (29)
Intermediate	105 (58)	111 (60)	45 (58)	27 (64)	113 (60)	401 (59)
High	29 (16)	16 (9)	7 (9)	2 (5)	26 (14)	80 (12)

* The intention-to-treat population included all participants who underwent randomization except the 1 participant who underwent randomization in error and was withdrawn immediately. Percentages may not total 100 because of rounding.

† Enrollment in the rifampin–clofazimine strategy group and the rifapentine–linezolid strategy group was discontinued before the full sample size was attained. Discontinuation of enrollment in these two strategy groups occurred before the sites in Uganda and India were opened.

‡ Body-mass index is the weight in kilograms divided by the square of the height in meters.

§ Sputum smears were not available for 2 participants. Smear grades were based on World Health Organization (WHO) guidelines. The highest grade from all smear examinations performed between screening and baseline is shown.

¶ Cycle threshold results were not available for 34 participants. The conversion of cycle threshold results from the Xpert MTB/RIF test (Cepheid) to estimates of bacillary burden was based on published consensus thresholds.

‖ The result from phenotypic susceptibility testing of the first available positive culture is shown. No participants had phenotypic resistance to rifampin.

** Low risk is defined as a negative smear and the absence of a cavity measuring more than 4 cm on a chest radiograph; intermediate risk as a positive smear of grade 2+ or lower and the absence of a cavity measuring more than 4 cm on a chest radiograph; and high risk as a positive smear of grade 3+, the presence of a cavity measuring more than 4 cm on a chest radiograph, or both. Relapse risk categories are based on the highest grade from all smear examinations performed and the largest cavity measurement on any chest radiograph obtained between screening and baseline. Two participants attempted but were unable to produce sputum at these study visits and were regarded as having a negative smear for the classification of relapse risk; neither of these participants had cavitation on a chest radiograph.

**Table 2:** Primary Efficacy Outcome.*

Outcome	Standard Treatment (N = 181)	Strategy with Rifampin–Linezolid (N = 184)	Strategy with Rifampin–Linezolid vs. Standard Treatment	Strategy with Bedaquiline–Linezolid (N = 189)	Strategy with Bedaquiline–Linezolid vs. Standard Treatment
			Adjusted Difference (97.5% CI)†		Adjusted Difference (97.5% CI)†
**Intention-to-treat population** ‡					
Primary outcome: composite of death, ongoing treatment, or active disease at wk 96 — no. (%)§	7 (3.9)	21 (11.4)	7.4 (1.7 to 13.2)	11 (5.8)	0.8 (−3.4 to 5.1)
Death before wk 96	2 (1.1)	5 (2.7)	—	1 (0.5)	—
Ongoing treatment at wk 96	2 (1.1)	8 (4.3)	—	5 (2.6)	—
Active disease at wk 96¶	1 (0.6)	4 (2.2)	—	3 (1.6)	—
Evaluation by telephone at wk 96 with no evidence of active disease but insufficient evidence of disease clearance when last seen	2 (1.1)	3 (1.6)	—	1 (0.5)	—
No evaluation at wk 96 and insufficient evidence of disease clearance when last seen	0	1 (0.5)	—	1 (0.5)	—
Outcomes classified as unassessable — no. (%)	1 (0.6)	1 (0.5)	—	2 (1.1)	—
Single positive culture at wk 96 but no other evidence of active disease‖	0	1 (0.5)	—	0	—
Death from a cause that was definitively unrelated to tuberculosis**	1 (0.6)	0	—	0	—
No evaluation at wk 96 and sufficient evidence of disease clearance when last seen	0	0	—	2 (1.1)	—
No primary outcome or outcome classified as unassessable — no. (%)	173 (95.6)	162 (88.0)	—	176 (93.1)	—
**Assessable population** ††					
Primary outcome — no./total no. (%)	7/180 (3.9)	21/183 (11.5)	7.5 (1.7 to 13.2)	11/187 (5.9)	0.8 (−3.4 to 5.1)
**Per-protocol population** ‡‡					
Primary outcome — no./total no. (%)	6/177 (3.4)	17/160 (10.6)	6.9 (0.9 to 12.8)	9/176 (5.1)	0.9 (−3.3 to 5.1)

* Results are shown for the standard-treatment group and the two strategy groups with complete enrollment; results for the two strategy groups with incomplete enrollment are shown in Tables S4 through S9.

† Differences were estimated with a generalized linear model with binomial distribution and adjustment for trial group, country, and relapse risk. Unadjusted results and results of Bayesian analysis are shown in Table S7. The 97.5% confidence intervals are shown to adjust for multiplicity. The differences are shown in percentage points.

‡ All 541 participants in the three groups with complete enrollment who were alive and undergoing follow-up were evaluated at week 96. Of these, 527 (97.4%) were evaluated in person and 14 (2.6%) by telephone. Of the participants who were evaluated in person, 522 (99.1%) underwent chest radiography, 505 (95.8%) produced at least one sputum sample that could be evaluated or were asymptomatic and unable to produce sputum (imputed negative), and 20 (3.8%) produced sputum samples that could not be evaluated.

§ The primary outcome was assessed with a prespecified algorithm (Section S10).

¶ All 8 participants had definitive active disease at week 96 with at least two positive sputum cultures. For each of these participants, whole-genome sequencing was performed on paired isolates obtained at week 96 and at baseline, and the week 96 strain was related to the baseline strain; this finding is consistent with relapse. There were no cases of presumptive or possible active disease at week 96.

‖ Whole-genome sequencing showed that the strain from the single positive culture at week 96 was related to the baseline strain.

** The cause of death was cervical cancer.

†† The assessable population included all participants in the intention-to-treat population except those who had an outcome that was classified as unassessable.

‡‡ The per-protocol population included all participants in the intention-to-treat population except those who did not complete the protocol-specified initial treatment or had inadequate treatment during the first 56 days, unless the reason for inadequate treatment was death.

**Table 3:** Secondary Outcomes.*

Outcome	Standard Treatment (N =181)	Strategy with Rifampin–Linezolid (N = 184)	Strategy with Rifampin–Linezolid vs. Standard Treatment	Strategy with Bedaquiline–Linezolid (N =189)	Strategy with Bedaquiline–Linezolid vs. Standard Treatment
			Difference (95% CI)†		Difference (95% CI)†
**Participant-centered outcomes**					
Total treatment time through wk 96 — days‡					
Total duration of treatment	180.2±37.9	105.7±80.1	−74.5 (−87.4 to −61.6)	84.8±65.3	−95.3 (−106.2 to −84.5)
Total qualifying treatment time	177.3±35.6	101.6±74.9	−75.7 (−87.7 to -−63.6)	83.8±64.2	−93.5 (−104.0 to −82.9)
Acceptability scores§					
Difficulty score	1.5±1.7	2.4±2.2	1.0 (0.6 to 1.4)	1.8±2.0	0.4 (0.0 to 0.8)
Anxiety score	3.6±2.2	3.9±2.0	0.3 (−0.1 to 0.8)	3.4±2.0	−0.2 (−0.6 to 0.3)
Motivation score	6.2±3.9	8.0±3.0	1.8 (1.1 to 2.5)	8.1±2.9	1.9 (1.2 to 2.6)
Preferred treatment recommendation to others — no. (%)					
2-Mo treatment	NA	126/176 (71.6)	—	141/180 (78.3)	—
6-Mo treatment	NA	35/176 (19.9)	—	25/180 (13.9)	—
No preference	NA	15/176 (8.5)	—	14/180 (7.8)	—
Quality-of-life scores¶					
Mental health summary score	57.5±0.5	57.5±0.5	−0.02 (−1.35 to 1.30)	57.8±0.5	0.33 (−1.02 to 1.68)
Physical health summary score	56.7±0.5	56.8±0.5	0.06 (−1.24 to 1.37)	56.7±0.5	0.00 (−1.25 to 1.26)
Health-status score‖	0.99±0.0	0.98±0.1	−0.01 (−0.02 to 0.00)	0.98±0.1	−0.01 (−0.02 to 0.01)
Body weight**					
Change from baseline — kg	5.8±4.8	5.6±4.7	−0.3 (−1.3 to 0.6)	6.1±4.8	0.2 (−0.7 to 1.2)
Change from baseline — %	11.9±10.0	11.4±9.8	−0.8 (−2.8 to 1.3)	12.1±9.8	0.3 (−1.7 to 2.4)
**Safety outcomes**					
Adverse events through wk 96 — no. (%)					
Any grade 3 or 4 adverse event	29 (16.0)	32 (17.4)	1.4 (−6.4 to 9.2)	30 (15.9)	−0.2 (−7.9 to 7.4)
Any serious adverse event	11 (6.1)	18 (9.8)	3.7 (−2.1 to 9.7)	14 (7.4)	1.3 (−4.2 to 6.9)
Death††	3 (1.7)	5 (2.7)	1.1 (−2.4 to 4.8)	1 (0.5)	−1.1 (−4.3 to 1.5)
Respiratory disability at wk 96 — no. (%)‡‡					
Grade on MRC breathlessness scale ≥3	0	2.7 (1.5)	1.5 (−0.5 to 3.5)	2.7 (1.4)	1.4 (−0.5 to 3.3)
FEV_1_ <50% of predicted value	24.3 (13.4)	20.5 (11.1)	−1.1 (−8.7 to 6.4)	22.4 (11.8)	0.1 (−7.8 to 7.9)
**Program-centered outcomes**					
Treatment adherence§§					
Adherence within first 56 days — % of days	98.8±5.5	95.9±10.0	−2.9 (−4.6 to 1.3)	98.4±6.6	−0.5 (−1.7 to 0.8)
Cessation within first 56 days — no. (%)	1 (0.6)	3 (1.6)	—	1 (0.5)	—
Acquired drug resistance — no. (%)¶¶	0	0	—	2 (1.1)	—
Relapse-associated transmission risk‖‖					
Transmission risk period — days	0.5±4.3	2.4±8.3	1.9 (0.5 to 3.2)	3.2±14.1	2.7 (0.6 to 4.8)
New exposed household contacts — no.	0.01±0.15	0.01±0.10	0.00 (−0.03 to 0.03)	0.06±0.40	0.05 (−0.01 to 0.10)

* Plus–minus values are means ±SD. Results are shown for the standard-treatment group and the two strategy groups with complete enrollment; results for the two strategy groups with incomplete enrollment are shown in Table S11. The approach to missing data for secondary outcomes is described in Section S13. FEV_1_ denotes forced expiratory volume in 1 second, MRC Medical Research Council, and NA not assessed.

† For total treatment time, acceptability scores, treatment adherence, and transmission risk, mean differences are shown. For quality-of-life scores, differences were estimated with a linear fixed-effects model with adjustment. For health-status scores and body weight, differences were estimated with a linear mixed model for repeated measures. For adverse events, differences in percentages are shown (in percentage points). For the MRC breathlessness scale outcome, differences were estimated with a logistic regression model with normal approximation to binomial distribution. For the FEV_1_ outcome, differences were estimated with a generalized linear model with binomial distribution.

‡ The total duration of treatment is defined as the total number of days from the first day through the last day of each treatment course for all courses received from baseline through week 96 for each participant. The total qualifying treatment time is defined as the total number of qualifying days from baseline through week 96. Qualifying days are defined as days on which the participant received at least 50% of the protocol-specified dose of all drugs in the regimen that was prescribed at that time. Details are provided in the protocol, which includes the statistical analysis plan.

§ Acceptability scores were derived from a study-specific questionnaire (Section S9 and Table S10). Scores range from 0 to 10, with higher scores indicating greater difficulty, anxiety, or motivation. Acceptability scores were not available for 21 participants (3.8%).

¶ Quality-of-life scores were derived from the Medical Outcomes Study HIV Health Survey. Summary scores range from 0 to 100, with higher scores indicating better quality of life. Quality-of-life scores were not available for 44 participants (7.9%).

‖ The health-status score is the European Quality of Life–5 Dimensions index score. Index scores range from less than 0 (worst) to 1 (best). Health-status scores were not available for 34 participants (6.1%).

** Body weight was not available for 40 participants (7.2%).

†† Causes of death were cervical carcinoma, cardiac arrest, and possible cerebrovascular accident (each in 1 participant) in the standard-treatment group; drug-induced liver injury, coronavirus disease 2019, and cirrhosis (each in 1 participant) and an unknown cause (in 2 participants) in the rifampin–linezolid strategy group; and tuberculosis in the bedaquiline–linezolid strategy group.

‡‡ The MRC breathlessness scale ranges from grade 1 to grade 5, with higher grades indicating a greater degree of activity-related breathlessness. Grades on the MRC breathlessness scale were not available for 40 participants (7.2%). FEV_1_ is measured with spirometry and is expressed as a percentage of the predicted value for a person of the same age, sex, height, and race (Section S7). FEV_1_ measurements were not available for 105 participants (19.0%) at week 8 and for 79 participants (14.2%) at week 96; missing values were imputed.

§§ Adherence within the first 56 days was based on the number of qualifying days from baseline through day 56. Cessation within the first 56 days refers to complete cessation of all drugs that started during the first 56 days and lasted for at least 56 consecutive days.

¶¶ Two participants in the bedaquiline–linezolid strategy group had confirmed acquired phenotypic resistance to bedaquiline (and clofazimine) that accompanied relapse at weeks 36 and 52; both had a 198 deletion in the *mmpR5* gene that was detected on whole-genome sequencing. Three cases of unconfirmed drug resistance that occurred in participants in the rifampin–linezolid strategy group are listed in Table S16. Phenotypic susceptibility testing was performed after all episodes of disease recurrence; at least one isolate was tested for susceptibility to all drugs associated with previous exposure in 93% of cases, for susceptibility to standard drugs only in 1% of cases, and for susceptibility to at least rifampin, isoniazid, and ethambutol in 5% of cases.

‖‖ The transmission risk period is defined as the time between the first smear of grade 1+ or higher obtained during a confirmed relapse episode and the time that retreatment is started or trial follow-up ends. New exposed household contacts are defined as persons living in the household during the transmission risk period who were not living there when the participant entered the trial.
