# Supplementary material for: Treatment Strategy for Rifampin-Susceptible Tuberculosis
Source: N Engl J Med. Author manuscript; Available in PMC 2024 Nov 25. (PMC7616851; doi:10.1056/NEJMoa2212537)
Supplement: Supplementary appendix [file EMS200036-supplement-Supplementary_appendix.pdf]

Supplementary Appendix

Supplement to: Paton NI, Cousins C, Suresh C, et al. Treatment strategy for rifampin-susceptible tuberculosis.
N Engl J Med 2023;388:873-87. DOI: 10.1056/NEJMoa2212537

This appendix has been provided by the authors to give readers additional information about the work.

TABLE OF CONTENTS

Members of the TRUNCATE-TB trial team ……………………………………………………………………………........4-6

Supplementary methods

Section S1 Complete eligibility criteria ……………………………………………………………………………......7-8

Section 2 Randomisation strata ………………………………………………………………………………………......9

Section 3 Treatment regimens …………………………………………………………………………………………….9-10

Section 4 Disease activity clinical management criteria ……………………………………………………….11-12

Section 5 Rationale for selection of arms for discontinuation of enrolment ………………………….…12

Section 6 Trial schedule …………………………………………………………………………………………………….13-14

Section 7 Assessment of respiratory disability ……………………………………………………………………..15

Section 8 Definition of drug resistance ………………………………………………………………………….…..16

Section 9 Assessment of acceptability ……………………………………………………………………………….16

Section 10 Primary outcome definition …………………………………………………………………………......17-22

Section 11 Justification of non-inferiority margin and projected proportion

with unsatisfactory outcome …………………………………………………………………………………………….23-24

Section 12 Analysis of the primary outcome ……………………………………………………………………….24

Section 13 Analyses of secondary outcomes and approach to missing data…..............................24-25

Supplementary tables

Table S1 Representativeness of study participants ………………………………………………………….26-27

Table S2 Initial treatment course, completion and switches ………………………………………………28-29

Table S3 Re-treatment courses ………………………………………………………………………………………….30

Table S4 Primary efficacy outcome, proportions at week 96
in the intention-to-treat population, all arms …………………………………………………………………….31-32

Table S5 Primary efficacy outcome, proportions at week 96
in the assessable population, all arms ……………………………………………………………………………….33

Table S6 Primary efficacy outcome, proportions at week 96
in the per-protocol population, all arms ………………………………………………………..…………………34

Table S7 Primary efficacy outcome, difference in proportions at week 96
In the intention-to-treat population, all arms….……………………………………………………………….35-36

Table S8 Primary efficacy outcome, difference in proportions at week 96
in the assessable population, all arms……………………………………………………………………………..37

Table S9 Primary efficacy outcome, difference in proportions at week 96
in the per-protocol population, all arms……...38

Table S10 Acceptability, all arms ……………………………………………………………………………………39

Table S11 Secondary outcomes, all arms …………………………………………………………………………….40-42

Table S12 Grade 3 and 4 and serious adverse events
between baseline and week 96, all arms ………………………………………………………………………43

Table S13 Grade 3 and 4 adverse events between baseline and week 96
by system organ class, all arms……………………………………………………………………………………………44-47

Table S14 Serious adverse events between baseline and week 96 by category, all arms …………48

Table S15 Grade 3 and 4 adverse events between baseline and week 96
by system organ class, for standard treatment arm and TRUNCATE strategy
(rifampicin-linezolid) arm, shown by period before and after rifampicin dose change…………….49

Table S16 Unconfirmed acquired drug resistance ………………………………..……………………………50

Supplementary figures

Figure S1 Change in body weight from baseline to week 96, all arms ……………………………………51

MEMBERS OF THE TRUNCATE-TB TRIAL TEAM

Participating Sites and central laboratories

Indonesia:
Rumah Sakit Umum Pusat (RSUP) Persahabatan, Jakarta (72): Erlina Burhan, Fathiyah Isbaniah, Ibrahim N.I.P. Dharmawan, Assica P.A. Hakiman, Hera Afidjati, Diadikma Belarosa, Aga Krisnanda, Nadia U.A. Hadi, Jihaan Hafirain, Dedy Aditia

Rumah Sakit Umum Daerah Dr. Soetomo, Surabaya (62): Tutik Kusmiati, Soedarsono Soedarsono, Deby Kusumaningrum, Ridwan Yasin, Nur S.R. Panenggak, Randy D. Kurniawan, Sri Rejeki, Novi Aryanti

Universitas Padjadjaran, Bandung (53): Rovina Ruslami, Prayudi Santoso, Alamanda Larasmanah, Naufal Ihsan, Yuanita Gunawan, Sheila Sumargo, Vycke Yunivita

RSUP Dr *Wahidin* Sudirohusodo, Makassar (52)*:* Irawaty Djaharuddin, Eliana Muis, Nurjannah Lihawa, Nasrum Massi, Yusfiana Majid, A. Siti Kahfiah Mukhlis, Imam Nurjaya, Siti Arifah Lacante

RSUD Saiful Anwar, Malang (50): Jani J.R. Sugiri**,** Gede Sasmika Suwandi**,** Kristo Kurniawan**,** Santony Santony**,** Herman Liem**,** Tiar Oktavian Effendi**,** Maria Kristiani, **,** Ni Made Rini

Rumah Sakit Umum Daerah (RSUD) dr. Moewardi, Solo (5): Jatu Aphridasari, Sandy Kurniawan, Dewi Astarini, Linda Soebroto

Balai Besar Laboratorium Kesehatan (BBLK) Surabaya (central laboratory): Titiek Sulistyowati;
BBLK Makassar (central laboratory): Yoeke Rasita;
FKUI Jakarta (central laboratory): Andriansjah Rukmana

Thailand:
Central Chest Institute of Thailand (28): Nonthaburi: Piamlarp Sangsayunh, Thanyanuch Sanchat, Phornchai Pingsusaen, Krisana Cheewakul, Sasithorn Bureechai, Waraporn Thuansuwan, Jirakan Boonyasopun, Karntheera Sangkaew, Sirijit Rattanawai

Chulalongkorn University, Bangkok (17): Anchalee Avihingsanon, Sivaporn Gatechompol, Hay Mar Su Lwin, Win Min Han, Thornthun Ureaphongsukkit, Prachya Chaiyahong, Pornmalai Suriya, Sasiwimol Ubolyam, Anuntaya Uanithirat, Apicha Mahanontharit, Plengsri Lertarrom

Taksin Hospital, Bangkok (4)*:* Supunnee Jirajariyavej, Stanrat Kanokdeeseerat, Kanyapat Wongwutcharajirakul

Philippines:
De La Salle Medical and Health Sciences Institute, Cavite (72): Victoria B. Dalay, Maria Marissa I. Golla, Emmanuel A. Gutierrez, Marietto L. Partosa, Genevieve V. Bayas, Cynthia G. Wagayen, Darecil B. Gelina, Eleonor S. Garcia, Angelita G. Pabruada, Laarean R. Perlas

Lung Center of the Philippines, Quezon City (58): Vincent M. Balanag, Jr., Nerissa A. Donato, Krizia Chloe R. Rivera, Paula Cindy M Villajuan, Zyra Zafe Del Rosario

Tropical Disease Foundation, Inc., Makati (42): Thelma E. Tupasi (deceased); Rholine Gem Martin Sindingan Veto, Maria Begonia Rejaba Baliwagan, Glenn Ibana Balane, Anthony A. Geronimo, Elsie Marie B. Dela Cruz

Perpetual Succour Hospital, Cebu (39): Anabella M. Guardiario, Maria Philina P. Villamor, Ma. Bernardita Sarcauga Chua, Peter Dela Torre Blanco, Rose Marie L. Cagwin, Karenza Antipuesto Camus

Philippine Tuberculosis Society Inc. - Quezon Institute, Quezon City (27): Jubert P. Benedicto, Ma. Kriselda Karlene G. Tan, Michelle B. Recana-Nieva, Rose Ann A. Espiritu-Villasfer

India:
National Institute of TB and Respiratory Diseases, New Delhi (14): Rohit Sarin, Jitendra Kumar Saini, Prabhpreet Sethi, Mohit Tomar, Manpreet Bhalla, Shivani, Arti, Shyam Singh Bisht

Uganda:
Infectious Diseases Institute, Kampala (41): Christine Sekaggya-Wiltshire, Ruth Mirembe Nabisere, Brian Otaalo, Jesca Asienzo, Letisha Najjemba, Juliet Nampala, Lucy Alinaitwe, Eunice Kaguiri

Joint Clinical Research Centre, Kampala (29): Cissy Kityo, Henry Mugerwa, Timothy Arthur Serumaga, Timothy Masaba, Theresa Najjuuko, Joseph Akol, Caroline Kayiza

Joint Clinical Research Centre, Mbarara (10): Abbas Lugemwa, Sharif Musumba, Ibrahim Yawe, Assumpta Katusiime, Beatrice Tumusiime, Mariam Kasozi, Myalo Sula, Rogers Ankunda

Trial coordination:
Yong Loo Lin School of Medicine, National University of Singapore: Nicholas Paton, Christopher Cousins, Celina Suresh, Nan Kai Ng, Elena Wan Yi Lur, Shariba Munawara, Felic Fanusi, Gail Cross, Anushia Panchalingham, Gianna Yau, Padmasayee Papineni, Kristina Rutkute, Meera Gurumurthy, Pauline Yoong

National University Hospital: Ka Lip Chew , Intan Permata Sari

Singapore Clinical Research Institute (SCRI): Qing Shu Lu, Shu Ling Lee, Mihir Gandhi, Yogesh Pokharkar, Rajesh Babu Moorakonda, Yin Bun Cheung

MRC Clinical Trials Unit at UCL: Angela Crook, Karen Sanders, Patrick Phillips, Andrew Nunn

London School of Hygiene and Tropical Medicine: Jody Phelan, Martin Hibberd

PRODIA, Indonesia: Catharina Aprillia

Syneos *Health* Indonesia: Amalia Rachmawati

Syneos Health Philippines: Larra Minnellie M. Esconde, Bianca Austria

HIV-NAT, Thailand: Kanitta Pussadee, Hathairat Prushyapornsri, Pornkhaun Mungklang, Chanapha Janpanich, Suzan Wilmott

JSS Medical Research, India: Taran Bedi

Trial Governance:
Trial Management Group: Nicholas Paton, Christopher Cousins, Celina Suresh, Padmasayee Papineni, Ibrahim Abubakar, Karen Sanders, Angela Crook, Andrew Nunn

Trial Steering Committee (TSC): Independent members: Geraint Davies (Chair), Charles Gilks, Sushil Pandey, Abdul Razak Bin Abdul Muttalif, Basanta Kumar Parajuli, Kaewta Sangsuk; Trial related members: Nicholas Paton, Erlina Burhan, Vincent Balanag, Anchalee Avihingsanon, Christine Sekaggya-Wiltshire, Rohit Sarin; Non-voting members: Angela Crook, Andrew Nunn

Independent Data Monitoring Committee (IDMC): Guy Thwaites (Chair), Matthew Law, Janice Caoili, Reinout Van Crevel

SUPPLEMENTARY METHODS

Section S1 COMPLETE ELIGIBILITY CRITERIA

Inclusion criteria

1. Age 18 to 65 years
2. Clinical symptoms consistent with pulmonary TB and/or evidence of pulmonary TB on CXR
3. Sputum GeneXpert test positive ¶
4. Willing to comply with the study visits and procedures
5. Resident at a fixed address that is readily accessible for visiting, within feasible travelling distance to the site and likely to remain resident there for the duration of trial follow-up
6. Willing to have directly observed therapy
7. Willing and able to provide written informed consent

Exclusion criteria

1. Taken more than 10 daily doses of standard anti-TB medication or fluoroquinolones during the 3 months prior to randomisation
2. Previous active TB disease for which treatment was given prior to the current episode (patients who have received isoniazid prophylaxis are eligible)
3. Known or suspected extra-pulmonary TB (in the opinion of the investigator; no specific screening tests required; if symptoms suggest extra-pulmonary involvement, patient can still be enrolled if relevant tests-performed for clinical management-exclude extra-pulmonary disease at the suspected site; pleural effusion occupying <50% of hemithorax or concomitant intra- or extra-thoracic lymphadenopathy are not exclusions)
4. Severe clinical pulmonary TB e.g. respiratory failure or complications likely to require hospital admission
5. Sputum smear 3+ on sample taken at screening (using WHO/IUATLD grading system)*
6. Cavity size > 4cm on screening CXR*
7. Presence of rifampicin resistance on GeneXpert test
8. Poorly-controlled diabetes that, in the opinion of the investigator, is unlikely to be controlled with available management strategies
9. Active malignancy requiring systemic chemotherapy or radiotherapy
10. Known Hepatitis B surface antigen positive and/or HCV antibody positive, unless liver function tests consistently within normal range for at least 2 years
11. History of myocardial infarction, congestive cardiac failure, cardiac arrhythmias or any known congenital cardiac problems
12. History of severe chronic lung disease (e.g. chronic obstructive pulmonary disease) with symptom score of ≥3 on MRC breathlessness scale
13. History of seizures*
14. Current tendinitis or history of tendinopathy associated with fluoroquinolone use*
15. Symptomatic peripheral neuropathy causing greater than minimal interference with usual social and functional activities.
16. Current alcohol or drug abuse
17. Women who are currently pregnant or breast-feeding
18. Women of childbearing potential unwilling or unable to use appropriate effective contraception (i.e. barrier methods such as condoms, hormonal contraception, intra-uterine device) for the first 6 months of the trial
19. Known allergy to one or more of the study drugs
20. Taking a concomitant medication that has a known or predicted interaction with any of the study drugs to which the patient might be randomised, or is known to prolong the QTc

interval (if the concomitant medication can be stopped after randomisation, or there is a feasible alternative medication available, the patient need not be excluded)

21. Taking any immunosuppressive drugs or use of systemic corticosteroids for more than 2 weeks prior to screening
22. Colour blindness detected by Ishihara test
23. 12-lead ECG at screening shows QTc greater than 450ms and/or any other clinically-significant abnormality such as arrhythmia or ischaemia
24. Any of the following laboratory parameters at screening:
    - Absolute neutrophil <1000 cells/µL, haemoglobin <7.0 g/dL, OR platelet count <50,000 cells/mm^3
    - Creatinine clearance of <60ml/min (calculated using Cockcroft-Gault equation,
    - ALT greater than 3 times the upper limit of normal
    - Uncorrected serum potassium <3.5 mmol/L
25. HIV antibody positive at screening*
26. Any other significant condition (e.g. psychiatric illness, chronic diarrhoeal disease), that would, in the opinion of the investigator, compromise the patient's safety or outcome in the trial or lead to poor compliance with study visits and protocol requirements
27. Participation in other clinical intervention trial or research protocol (participation in other studies that do not involve an intervention may be allowed, but this must be discussed and approved by the Chief Investigator)

*Criteria marked with an asterisk (5,6,13, 14) were removed in version 2 of the protocol.

Criterion 25 (HIV antibody positive), was modified in version 2 of the protocol as follows:
HIV antibody positive, unless **all** the following additional criteria are met:

(i) Current CD4 T-cell count >200cells/mm^3
(ii) Not currently taking ART
(iii) Patient and doctor willing to defer starting ART until 8 weeks after enrolment (up to 12 weeks, if randomised to a boosted regimen the duration of which is extended)
(iv) have access to ART and are willing to start when recommended by local treatment guidelines, at any point from 8 weeks (or 12 weeks, as above) after enrolment

Version 2 of the protocol was implemented, with removal of eligibility restrictions above, following approval of ethics committees and national regulatory agencies at 11 sites in Indonesia and Philippines. Approvals were received between December 2019 and February 2020, a mean of 9 weeks prior to end of trial enrollment.

¶ GeneXpert test: results of either Xpert MTB/RIF or Xpert Ultra were acceptable.

Section S2 RANDOMISATION STRATA

Randomisation was stratified by the following factors:

- Site
- Relapse risk
    - Lower risk: sputum screening smear negative and screening CXR cavities ≤ 4 cm and HIV negative
    - Intermediate risk: screening sputum smear positive and screening CXR cavities ≤ 4 cm and HIV negative
    - Higher risk: screening sputum smear grade 3+ or screening CXR cavities > 4 cm or HIV positive

The threshold of 4cm for cavitation on chest radiograph was based on studies of exposure-response relationships in rifapentine Phase II trials – Savic, Clin Pharmacol Ther 2017; 102(2):321-331.

Participants unable to produce sputum at screening were regarded as smear negative for the purpose of classifying relapse risk.

Section S3 TREATMENT REGIMENS

A. Standard treatment arm

For first 8 weeks

DRUG	<40KG	40-54 KG	55-70KG	≥71KG
Rifampicin	300mg	450mg	600mg	750mg
Isoniazid	150mg	225mg	300mg	375mg
Pyrazinamide	800mg	1200mg	1600mg	2000mg
Ethambutol	550mg	825mg	1100mg	1375mg

For subsequent weeks to week 24

DRUG	<40KG	40-54 KG	55-70KG	≥71KG
Rifampicin	300mg	450mg	600mg	750mg
Isoniazid	150mg	225mg	300mg	375mg

B. Rifampicin-linezolid arm
For 8 weeks

DRUG	<40KG	40KG- 54KG	55KG - 70KG	≥71KG
Rifampicin	35mg/kg (rounded to nearest 150mg, maximum 2100mg)*			
Isoniazid	150mg	225mg	300mg	375mg
Pyrazinamide	800mg	1200mg	1600mg	2000mg
Ethambutol	550mg	825mg	1100mg	1375mg
Linezolid	600mg			

*The dose of rifampicin was decreased from 35mg/kg to 20mg/kg for all participants from 1st November 2019 after 88 participants had been enrolled in this arm (recommendation of the Trial Steering Committee as a precaution following a death from drug-induced liver injury in this arm).

C. Rifampicin-clofazimine arm

For 8 weeks

DRUG	<40KG	40KG- 54KG	55KG - 70KG	≥71KG
Rifampicin	35mg/kg (rounded to nearest 150mg, maximum 2100mg)			
Isoniazid	150mg	225mg	300mg	375mg
Pyrazinamide	800mg	1200mg	1600mg	2000mg
Ethambutol	550mg	825mg	1100mg	1375mg
Clofazimine	200mg			

D. Rifapentine-linezolid arm

For 8 weeks

DRUG	<40KG	40KG- 54KG	55KG - 70KG	≥71KG
Isoniazid	5mg/kg rounded to nearest 100mg		300mg	
Pyrazinamide	25mg/kg rounded to nearest 500mg	1000mg	1500mg	2000mg
Rifapentine	1200mg			
Linezolid	600mg			
Levofloxacin	1000mg			

E. Bedaquiline-linezolid arm

For 8 weeks

DRUG	<40KG	40KG- 54KG	55KG - 70KG	≥71KG
Bedaquiline	400 mg once daily for 2 weeks then 200mg three times a week			
Isoniazid	5mg/kg rounded to nearest 100mg		300mg	
Pyrazinamide	25mg/kg rounded to nearest 500mg	1000mg	1500mg	2000mg
Ethambutol	15mg/kg (rounded to nearest 100mg, maximum 1600mg)			
Linezolid	600mg			

The rationale for selection of drugs and doses for the regimens is described in the trial protocol.

Section S4 DISEASE ACTIVITY CLINICAL MANAGEMENT CRITERIA

These criteria were used for real-time diagnosis of clinical relapse and re-treatment decisions

Patients were considered to have met criteria for clinical disease activity (clinical relapse) if at least 2 out of criteria A or B or C are satisfied, or if criterion D is satisfied. The criteria needed to be satisfied simultaneously and be based on recent imaging and microbiological investigations.

A. Clinical Disease Progression

New, recurrent or increased severity of one or more standard TB-related symptoms (cough, one or more episodes of haemoptysis, fever, pleuritic chest pain, weight loss, night sweats) or physical signs compared with the end of the last course of TB treatment (or period after the end of treatment if further improvement occurred subsequently) without alternative explanation(s) considered more likely in the opinion of the managing clinician.

B. CXR Progression

Presence of abnormalities that were compatible with active TB disease (cavitation, infiltrates, consolidation) with clear evidence of progression compared with CXR at end of the last course of treatment (or period after the end of treatment if further improvement occurred subsequently) without alternative explanation(s) considered more likely in the opinion of the managing clinician.

C. Microbiological persistence / progression

Sputum sample taken at or after the end of treatment that was:
Smear positive OR
GeneXpert positive (if >24w after end of treatment; or if end of treatment test was negative) OR
Culture positive

D. Confirmed positive sputum culture

Sputum culture positive on two consecutive samples taken on separate days with at least one taken ≥ 4 weeks after end of treatment**.**

<u>Extra-pulmonary TB disease activity</u>

The above criteria applied to relapse from pulmonary TB. The definition of relapse could also be met if the patient fulfilled equivalent criteria that indicate disease relapse at another site i.e. non-pulmonary symptoms or signs, evidence of abnormalities on another imaging test that were compatible with active TB disease with evidence of progression from an earlier comparable imaging test (if no previous test available for determining progression, the abnormalities should be of sufficient severity to explain the symptoms) and microbiological evidence (culture evidence, preferred). Histological evidence considered to show characteristic changes of TB would also be acceptable under criterion C. However, the evidence from A, B and C (or the two cultures for D) should relate to the same disease site.

Section S5 RATIONALE FOR SELECTION OF ARMS FOR DISCONTINUATION OF ENROLMENT

The planned interim analyses, which represent the phase 2 component of the seamless phase 2 / 3 trial design, are described in detail in the Protocol, section 10.4.

In summary, the Independent Data Monitoring Committee (IDMC) performed two pre-specified interim analyses at which they determined whether arms should discontinue enrollment based on the following thresholds:

> First interim analysis (after 30 participants in control arm reached 6 months post-randomisation): combined treatment failure/relapse rate above 25%, or hazard ratio of time to stable culture conversion versus control not exceeding 0.8
>
> Second interim analysis (after 70 participants in control arm reached 6 months post-randomisation): combined treatment failure/relapse rate above 20%, or hazard ratio of time to stable culture conversion versus control not exceeding 0.9.

The thresholds for these interim analyses were chosen to ensure high power for identifying arms that correspond to desirable treatment strategies but stopping recruitment to arms that are likely to have high rates of treatment failure or relapse. Simulation studies showed that the maximum family-wise error rate (the type I error under the assumption that none of the arms are non-inferior but no arms are dropped at interim analysis) is maintained at 4%.

The simulation studies are described in Protocol section 10.3 and the approach is explained in the following reference:

Bratton et al, Trials (2016) 17: 309. Type I error rates of muti-arm multi-stage clinical trials: strong control and impact of intermediate outcomes.

Based on the results at interim analyses, the IDMC did not recommend discontinuation of enrollment to any arm. However, two arms were selected by the Trial Steering Committee to discontinue enrollment early. The selection was made on pragmatic grounds, blinded to outcome data. One arm was selected for discontinuation following each of the two IDMC meetings.

The TRUNCATE strategy rifapentine-linezolid arm was selected to discontinue enrollment (after 42 were enrolled) mainly due to the relatively high pill burden compared to other regimens. For participants in the 40-54kg weight band (the majority), 15 pills per day were required for this regimen, in contrast to 9-10 pills per day for the other regimens (12 per day for the first 14 days in the bedaquiline-linezolid regimen). The decision was also influenced by new regulatory guidance on quinolone toxicity.

The TRUNCATE strategy rifampicin-clofazimine arm was selected to discontinue enrollment (after 78 were enrolled) mainly due to refusal by one national regulator to allow clofazimine importation.

Section S6 TRIAL SCHEDULE

VISIT TIMING[1]	SCREENING	D0	W1	W2	W4	W6	W8	W10	W12	W16	W20	W24	W36	W48	W60	W72	W84	W96
Informed Consent	X																	
Eligibility criteria	X	X																
Randomisation		X																
CLINICAL EVALUATION																		
Medical history & demographics	X	X																
Symptoms	X	X	X	X	X	X	X	X	X	X	X	X	X	X	X	X	X	X
Physical examination	X	X	X	X	X	X	X	X	X	X	X	X	X	X	X	X	X	X
Medication review and adherence	X	X	X	X	X	X	X	X	X	X	X	X	X	X	X	X	X	X
HEALTHCARE UTILISATION & QOL																		
Healthcare utilisation		X	X	X	X	X	X	X	X	X	X	X	X	X	X	X	X	X
EQ-5D		X	X	X	X	X	X	X	X	X	X	X	X	X	X	X	X	X
MOS-HIV		X																X
Patient acceptability questionnaire														X				X
Socioeconomic evaluation		X																X
INVESTIGATIONS																		
ECG[2]	X	X	X		X		X											
CXR[3]	X	X					X											X
Spirometry							X							X				X
URINE																		
Pregnancy test	X				X		X											
Urine for storage[4]		X			X		X					X						
SPUTUM																		
Smear[5]	X	X	X	X	X	X	X	X	X	X	X	X	X	X	X	X	X	X
Liquid culture[6]		X	X	X	X	X	X	X	X	X	X	X	X	X	X	X	X	X
GeneXpert test[7]	X						X											
Drug susceptibility tests[8]		X					X											
BLOOD																		
Standard safety monitoring[9]	X	X	X	X[10]	X	X[10]	X	X[10]										
HIV test[11] (and CD4 count)[12]	X																	
Drug levels (PK)[13]		X			X		X					X						
Plasma and RNA storage[14]		X			X		X					X						

1. Additional telephone visits at weeks 30, 40, 44, 52, 56, 64, 68, 76, 80, 88, 92 (omitted if attended scheduled visit within previous 7 days) to evaluate symptoms, medication and adherence. A telephone visit will be performed at the time of trial closure. Visit windows: ±3 days for W1 to W4; ± 5 days for W6 and W8; ± 7 days for W10 to W24; ± 14 days for W30 onwards and ± 28 days for W96.
2. Screening ECG may be deferred to Day 0, but result must be available prior to performing randomisation. Additional ECG required at end of treatment for patients who stop boosted regimen between week 8 and 12; additional ECG required at week 12 for patients who switch boosted regimen to standard treatment between week 8 and 12. If QTc >450 ms, ECG should be repeated to confirm.
3. CXR at any visit need not be repeated if done in previous 7 days and film is available for evaluation. Additional CXR done at end of treatment and at the first visit of any suspected episode of relapse.
4. Urine (10ml) for storage. Additional sample taken for storage and for pregnancy test at end of treatment and at first suspicion of relapse (one for each episode)
5. 2 sputum smears on separate days at week 96.
6. Spot sputum sample at each visit (if available), cultured on liquid media (MGIT); 2 sputum samples on separate days at week 96.
7. GeneXpert test at screening need not be repeated if a positive result is available from a test performed earlier during this illness episode, done in study-approved laboratory and results are/will be available to research team. GeneXpert at week 8 (and end of treatment, if treatment continues after 8 weeks), and on the initial sputum sample obtained during any suspected episode of relapse.
8. Drug susceptibility at baseline, and on positive cultures at or after week 8 (monthly during any retreatment episode).
9. Blood for standard safety monitoring: FBC, electrolytes (sodium, potassium, creatinine), LFTs (ALT, alkaline phosphatase, bilirubin) done at scheduled visits and at end-of-treatment visit. Additional bloods at screening only: amylase/lipase, magnesium, calcium and glucose. Blood tests at any visit need not be repeated if results available from previous 2 days.
10. Additional FBC at weeks 2, 6, and 10 for patients receiving linezolid (Arms B, D, E only).
11. HIV antibody test need not be repeated if done in previous 30 days and results available to trial team.
12. CD4 count only if patient HIV positive and trial is open to enrolment of HIV-positive participants at that site.
13. Blood for drug levels to be taken at Day 0 (1 hour post-dose), week 4 (pre-dose and 2 hours post dose) and week 8 (pre-dose and 4 hours post-dose), and week 24 (patients randomised to bedaquiline or clofazimine arms only, single sample, no timing requirements). Samples may be omitted for patient convenience or logistical reasons.
14. Blood for plasma (10ml EDTA tube) and host RNA (5ml RNA preservation tube) storage. Additional samples taken at end of treatment and at first suspicion of relapse (one for each episode).

Section S7 ASSESSMENT OF RESPIRATORY DISABILITY

Respiratory disability was assessed at week 96 in two ways:

(i) MRC Breathlessness Scale
Patient self-reported breathlessness was assessed using the MRC Breathlessness scale, with respiratory disability defined as a score of ≥3. Assessment of respiratory disability using the breathlessness scale was also done at baseline, end of treatment, weeks 48 and 96 (data not used for the present analysis other than for imputation of missing values at week 96; see section S13).

(ii) Spirometry
Spirometry was performed using a hand-held spirometer, standardised across all sites (EasyOne Air). Measurements were performed at week 8, week 48 and week 96. The initial spirometry assessment was performed at week 8, rather than the baseline visit, for the following reasons:

- difficulty of obtaining a reliable spirometry measurement when patients are acutely unwell
- guideline recommendations (British Thoracic Society) to defer spirometry in people with newly diagnosed tuberculosis until at least 2 weeks after the start of treatment
- the substantial burden of essential tests and processes at the baseline visit for participants and staff; and the potential negative impact on the time available for and resultant quality of these baseline tests and processes (including time to discuss with the participant the regimen and establish an approach to supervision of treatment) if spirometry were to be added to the visit requirements
- the likely greater utility of a week 8 measurement as a reference point for assessing any impact of the strategy on lung function; the hypothesis is that any strategy-related lung damage would be driven by relapses that can only happen after week 8; recovery in lung function from baseline to week 8 would serve mainly to mask any subsequent strategy-related changes if baseline were taken as the reference point for change at week 96.

Assessment of FEV1 was graded based on number of acceptable measurements and reproducibility of the two highest acceptable measurements of each parameter, using the American Thoracic Society/ European Respiratory Society 2019 guidelines. At least one acceptable measurement was required (Grade E) for a participant assessment to be included at a particular timepoint.

Maximum FEV1 was expressed as a percentage of the predicted value for an individual of that age, sex, height, and race (FEV1%) calculated using the Global Lung Function Initiative (GLI) 2012 equations. [Quanjer PH et al; Multi-ethnic reference values for spirometry for the 3-95-yr age range: the global lung function 2012 equations. Eur Respir J. 2012;40(6):1324–43].

Respiratory disability was defined as FEV1% < 50% at week 96 (i.e. severe or very severe on GOLD criteria).[Vestbo J et al. Global strategy for the diagnosis, management, and prevention of chronic obstructive pulmonary disease: GOLD executive summary. Am J Resp Crit Care Med 2013; 187(4): 34]. New respiratory disability at week 96 was defined as respiratory disability present at week 96 that was not present at week 8 (i.e. FEV1% was ≥ 50% at week 8).

Additional details are provided in the statistical analysis plan. The approach for handling missing data on spirometry is described in section S13.

Section S8 DEFINITION OF DRUG RESISTANCE

The primary definition of drug resistance is based on phenotypic drug resistance testing, with whole genome sequencing (and other relevant molecular tests that were done) considered as supporting data.

Phenotypic resistance on a single isolate was ignored if the same isolate was shown to be susceptible on repeat testing; or if the isolate was followed by one or more isolates that were shown to be susceptible.

<u>Baseline drug resistance</u>

Participants were classified as having baseline drug resistance to a specific drug if there was demonstration of phenotypic resistance to that drug on the first available isolate, obtained at or before week 2 of the trial. If the first available isolate was after week two, drugs to which the isolate was shown to be susceptible were inferred as susceptible at baseline; drugs that were shown to be resistant after week two were designated as unknown susceptibility at baseline.

<u>Acquired drug resistance</u>

Participants were classified as having acquired drug resistance (confirmed) to a specific drug if there was demonstration of phenotypic resistance to that drug on at least two separate isolates during follow-up; and demonstration of phenotypic susceptibility to that drug on two separate samples collected prior to the first of the samples showing resistance; and exposure to the relevant drug (or another drug known to cause cross-resistance to that drug) prior to the collection of the first sample showing resistance.

Participants were classified as having acquired drug resistance (unconfirmed) if there was demonstration of resistance on one follow-up isolate only (without subsequent susceptible isolates); or susceptibility on one previous isolate only; or where there was no exposure to the relevant drug.

Cases of acquired drug resistance were rejected if results from molecular tests or whole genome sequencing showed resistance mutations consistent with the phenotypic drug resistance were present at baseline; or where they show that the follow-up resistant strain was not related to the earlier susceptible strain.

Additional details are provided in the statistical analysis plan.

SECTION S9 ASSESSMENT OF ACCEPTABILITY

Acceptability was assessed using a study-specific questionnaire administered at Weeks 48 and 96 to all participants. Questions asked about how much difficulty (swallowing pills, attending post-treatment clinic visits) or anxiety (risk of side effects, recurrence of tuberculosis or infecting others) participants had experienced, answered as “none”, “a little”, “some” or “a lot” at week 48 and 96. Responses within the difficulty and anxiety domains were averaged and normalized to a scale of 10 (0, “none”; 10, “a lot”).

Motivation was assessed by a question on how much the planned treatment duration increased adherence motivation when starting treatment, analysed based on the response at week 48 only. Overall acceptability was assessed by a question about which treatment option the participant would recommend to a friend, analysed based on the response at week 96 only.

Additional details are provided in the statistical analysis plan.

Section S10 PRIMARY OUTCOME DEFINITION

Details of the primary outcome are provided in the statistical analysis plan (sections 4.11 and 4.12).

The difference between (and rationale for) unsatisfactory outcome used for this strategy analysis and treatment failure/relapse (or unfavourable outcome) used for conventional phase 3 regimen efficacy analyses is explained in part 5 of this section, below. A side-by-side comparison of the two outcomes (unsatisfactory outcome versus unfavourable outcome) is provided in a table in the statistical analysis plan (section 4.13).

The primary outcome is unsatisfactory clinical outcome at week 96. This is a composite outcome chosen to represent a pragmatic view of the outcome of the management strategies from the patient and programme perspective.

This composite outcome is derived from a classification on the following 3 components:

- Ongoing requirement for TB treatment at week 96
- Ongoing TB disease activity at week 96
- Death prior to week 96

Each of these components is classified as unsatisfactory or unassessable at week 96. If any of the 3 components are classified as *unsatisfactory* the overall clinical outcome will be classified as *unsatisfactory*. If none of the components are classified as unsatisfactory but one or more of the components are classified as *unassessable*, then the overall clinical outcome will be classified as *unassessable*. If none of three components are classified as unsatisfactory or unassessable then the overall clinical outcome will be classified as *satisfactory*.

1) Classification of clinical outcome for patients who attend during the week 96 analysis window or are known to have died

<u>(i) Ongoing requirement for TB treatment at week 96</u>

Classify as unsatisfactory if the following are met:

- Is taking any combination of TB drugs when seen during the week 96 analysis window.
  [For avoidance of doubt, this requires simply that the patient is on treatment at week 96; there is no requirement to have met the diagnostic criteria at the start of the treatment course].

OR

- Has been prescribed a course a course of treatment for a new episode of active TB (meeting the disease activity clinical management criteria, defined as above) starting within 6 months (24 weeks) prior to the scheduled week 96 visit date (i.e. after week 72, day 504) but has stopped the drugs before the week 96 visit. If has been prescribed treatment but did not meet disease activity clinical management criteria at the start of treatment (or it is not known whether met those criteria at the start of treatment) classify as unassessable on this outcome.

(ii) Ongoing TB disease activity at week 96

Classified by disease activity research criteria (defined below), with some modifications based on WGS results and the presence of negative cultures.

If only criteria A and B are met (not C or D), the classification of unsatisfactory outcome will be changed to unassessable if there is ≥ 1 negative sputum sample (observed) in the week 96 analysis window.

If only criterion C is met (not A, B or D), disease activity research criteria are not met. This special case - isolated positive sputum culture – will result in a classification of unassessable, unless EITHER WGS at week 96 shows a different strain from baseline OR WGS is inconclusive or not done and there is a second culture within the week 96 analysis window at which an observed negative value is obtained (not imputed): in both of these scenarios the isolated positive culture will be ignored.

(iii) Death during or prior to week 96 analysis visit window

All deaths during or prior to the close of the week 96 analysis window will be classified as an unsatisfactory outcome, with the following exception:

If the patient had a study visit at or after week 48; was not on treatment and had no evidence of ongoing TB activity when last seen (in study clinic or home visit; ongoing TB activity assessed by *disease activity clinical management criteria*); and the cause of death is known to be unrelated to TB or the drugs used to treat it (e.g. road traffic accident; cause of death will be taken to be unrelated to TB or drugs based on the SAE form indicating "unrelated" to both) the patient will be classified as unassessable on this outcome.

2) Classification of clinical outcome for patients not attending during the week 96 analysis window

If the patient did not attend in person during the week 96 window (and was not seen for a home visit), information gathered at a telephone visit with the patient may be used to make an evaluation. If there is no in-person patient visit or patient telephone visit within the week 96 analysis window (to week 108), information gathered from friend/relative telephone visits within the week 96 analysis window (week 90 to 108) will be considered in the final outcome.

Deaths will only be counted in the primary outcome analysis if the date of death occurs before the close of the week 96 analysis window. Any report of patient being alive at week 96 will only be considered if there is a report of a friend/relative seeing the patient in person on a date on or after the opening of the week 96 analysis window (i.e. from week 90). Any report of a patient being on treatment or having symptoms will only be considered if the friend/relative reports seeing the patient in person on a date within the week 96 analysis window (considered to be within the first day of the first month to the last day of the last month covered by the analysis window, if the friend/relative can only estimate the date to the nearest month).

Classification will be based on the following rules.

If the patient:

(a) Completed their last prescribed course of treatment (minimum 54 days of the boosted regimen, or 154 qualifying treatment days if on or switched to standard regimen) AND

(b) Had at least one study visit at (on the day of cessation) or after completing the course of treatment AND

(c) Did not meet the criteria for ongoing requirement for treatment or ongoing TB disease activity when last seen (assessed by disease activity clinical management criteria, above); evidence of CXR progression omitted unless CXR available at the visit when last seen); AND

(d) The last two sputum cultures obtained at study visits, taken at or following the completion of treatment ("at" defined as no more than 7 days prior to cessation of treatment) were negative (observed; or imputed from inability to produce sputum, with clinical disease recovery or by meeting criteria for sputum omission) AND

(e) EITHER
The patient can be contacted at week 96 and confirms that they are not currently taking TB treatment (or have been prescribed TB treatment that they should be taking – information on intent to prescribe at last attended visit takes precedence over information provided by the patient), AND has no current pulmonary symptoms meeting the criteria of clinical evidence of TB (as listed in disease activity clinical management criteria), discounting symptoms clearly attributable to other causes)
OR
the patient cannot be contacted but a designated friend / relative verifies that they have personally seen the patient (within the week 96 visit analysis window) and that the patient was alive, neither reported taking TB treatment nor that they had symptoms meeting the criteria of clinical evidence of pulmonary TB (above) nor were observed to have symptoms meeting such criteria.

If (a) to (e) all true, classify as satisfactory clinical outcome.

If (a) to (d) all true, but there is no information available from patient or friend/relative to make a judgement on (e) in the absence of the patient having been contacted, classify as unassessable.

If (a) to (e) are not all true, classify as unsatisfactory.

Where additional information is available for a patient at week 96 (such as smears of CXR done at local clinics or the site clinic) but the patient is not seen in person (at clinic or home visit), the patient will be evaluated under the criteria in this section. The additional information will not alter the assessment of the outcome, but may be reported as supporting information for the verdict derived from the above decision rules.

3) Disease activity research criteria

These criteria are used to determine the *ongoing disease activity* component of the primary outcome at week 96 (section 1, (ii) above). The criteria are assessed based on the clinical status at the definitive week 96 visit and the tests performed at that visit. The definitive week 96 visit is the

first in-person visit within the protocol-mandated visit window (week 92 to week 100); or if there is no visit within the protocol-mandated visit window, then use the first in-person visit within the analysis visit window (week 90 to week 108).

Where the clinical status at the week 96 visit or the results of tests performed at the week 96 visit indicate a possibility of relapse and where a formal relapse assessment is then performed, the results of any assessments (clinical, imaging, microbiology) performed as part of the relapse assessment will be considered in the evaluation of the disease activity at week 96, provided that they were done within the week 96 analysis window.

The disease activity research criteria are classified as *met* if:

- At least 2 out of criteria A or B or C are satisfied
  OR
- Criterion D is satisfied.

The strength of evidence for ongoing disease activity is further classified as:

- Definitive: Criterion D is satisfied (with or without other criteria)
- Presumed: Criterion C is satisfied with at least one of A or B satisfied
- Possible: Criterion A and B satisfied only

Criteria:

A. Clinical evidence:

New, recurrent, or increased severity of one or more standard TB-related symptoms (cough, one or more episodes of haemoptysis, fever, pleuritic chest pain, weight loss, night sweats) for a duration of at least 7 days, or of physical signs that are suggestive of ongoing TB disease activity/ relapse, compared with the end of the last course of TB treatment (or period after the end of treatment if further improvement occurred subsequently) without alternative explanation(s) considered more likely in the opinion of the managing clinician.

B. CXR evidence

CXR taken within week 96 analysis window that has abnormalities that are compatible with active TB disease (cavitation, infiltrates, consolidation) and clear evidence of progression compared with CXR at the end of the last course of TB treatment (or period after the end of treatment if further improvement occurred subsequently) without alternative explanation(s) considered more likely in the opinion of the managing clinician.

C. Microbiological evidence

Observed sputum culture positive for *Mycobacterium tuberculosis* on 1 sample taken within the week 96 analysis window.

D. Microbiological evidence

2 positive cultures: Sputum culture positive for *Mycobacterium tuberculosis* on 2 samples taken on separate days with at least one taken within the week 96 analysis window [if only one positive sample in the week 96 window, examine the week 84 window for confirmatory sample]

OR

One positive culture plus other: Sputum culture positive on 1 sample taken within the week 96 analysis window AND a second sample taken on a separate day within the week 96 analysis window that is either:

Smear positive (only if taken > 12 weeks after the end of the last course of treatment) and/or

GeneXpert positive (only if taken > 24 weeks after the end of treatment or if end of treatment result was negative).

Extrapulmonary TB disease activity

The above criteria apply to evidence of ongoing pulmonary TB disease activity. The disease activity research criteria may also be met at week 96 if the patient fulfils equivalent criteria that indicate ongoing TB disease activity at another site i.e. non-pulmonary symptoms or signs, evidence of abnormalities on another imaging test that are compatible with active TB disease with evidence of progression from an earlier comparable imaging test (if no previous test available for determining progression, the abnormalities should be of sufficient severity to explain the symptoms), and microbiological evidence (presumptive or definitive) based on samples obtained from another site. However, the evidence from A, B and C (or the two samples positive for D) should relate to the same disease site.

Disease activity modified by whole genome sequencing

Whole genome sequencing (WGS) will be performed, where possible, in all new episodes of TB disease activity diagnosed throughout the trial and used to interpret whether the disease activity represents relapse of reinfection.

4) Summary of algorithm for classification of clinical outcome at week 96 for analysis of the strategy

**EVENT**	**Classification of clinical outcome at week 96**
Ongoing requirement for TB treatment at week 96	
Started treatment at or after week 72 but stopped treatment before week 96<br>Exception: Did not meet *disease activity clinical management criteria* when started treatment	Unsatisfactory<br><br>Exception: Unassessable
On treatment at week 96	Unsatisfactory
Ongoing TB disease activity at week 96	
Assessed by *disease activity research criteria* at week 96:<br>*Definitive* (D) or *Presumed* (C + A and/or B) disease activity<br>[WGS same strain, inconclusive or not done]<br>Exception: WGS shows different strain from baseline	Unsatisfactory<br><br><br>Exception: Unassessable
Assessed by *disease activity research criteria* at week 96:<br>*Possible* (A+B only) disease activity	Unsatisfactory

Exception: At least one sputum culture (observed) negative at week 96	Exception: Unassessable
Single positive culture at week 96	
Assessed by *disease activity research criteria* at week 96:<br>Criterion C met (A, B and D all not met)<br>WGS same strain; or WGS inconclusive or not done<br>Exception 1: WGS shows different strain from baseline<br>Exception 2: WGS inconclusive or not done and at least one other sputum culture (observed) negative at week 96	Unassessable<br><br><br>Exception 1: Ignore<br>Exception 2: Ignore
Death at or prior to week 96	
Death before close of week 96 analysis window<br>Exception:<br>Last study visit was after week 48 AND well, not on treatment, no evidence ongoing TB activity at last study visit [assessed by *disease activity clinical management criteria*] AND cause of death known to be unrelated to TB or TB drugs	Unsatisfactory<br><br>Exception: Unassessable
Did not attend week 96 visit (and not known to have died)	
Withdrawal, lost to follow up, missed week 96 visit<br>Exception 1:<br>(a) Completed last prescribed course of treatment (minimum 54 days of boosted regimen, or 154 qualifying treatment days if on or switched to standard regimen) AND<br>(b) Had at least 1 study visit at or after completing last course of treatment AND<br>(c) Did not meet the criteria for ongoing requirement for treatment [addressed by (a), above] or ongoing TB disease activity when last seen [assessed by *disease activity clinical management criteria*; evidence of CXR progression omitted unless CXR available at the visit when last seen] AND<br>(d) Last 2 visits (at or following end of treatment) had negative sputum cultures (observed or imputed based on clinical recovery)<br>AND<br>(e) EITHER patient can be contacted at week 96 and confirms not on treatment and no TB symptoms OR friend/relative can be contacted at week 96 confirming seen alive in previous 2 months, did not report TB treatment nor symptoms (or observed to have symptoms)<br>(a) to (e) all true<br>Exception 2:<br>(a) to (d) all true, no information available to judge (e)	Unsatisfactory<br><br>Exception 1: Ignore<br><br><br><br><br><br><br><br><br><br><br><br><br><br><br><br>Exception 2:<br>Unassessable

5) Difference between unsatisfactory outcome used for evaluation of the strategy and conventional outcomes used for regimen comparisons in phase 3 trials

Although relapses (or treatment failure) occurring during follow-up have traditionally formed the basis of the evaluation of regimen efficacy in conventional phase 3 trials, these events do not affect the primary outcome of the strategy analysis if re-treatment has been completed and the participant does not have active disease at week 96. This is because short initial treatment followed by observation for, and retreatment of, relapses form an integral part of the management strategy that is being evaluated in the trial. The strategy cannot be properly evaluated if its implementation is counted as an unsatisfactory outcome on the primary efficacy analysis.

Section S11 JUSTIFICATION OF NON-INFERIORITY MARGIN AND PROJECTED PROPORTION WITH UNSATISFACTORY OUTCOME

A non-inferiority margin of 12% was considered to be an acceptable difference to clinical collaborators, given the expected large reduction in total treatment duration.
This margin is also consistent with FDA guidance. It is estimated that the case fatality rate for untreated smear positive pulmonary tuberculosis is 70%, ie a cure rate of 30% (Tiemersma et al, 2011). If we assume 90% satisfactory outcomes after two years on standard treatment, this gives a standard treatment effect (denoted as M1 in FDA guidance document) of 60%. Selecting a non-inferiority margin of 12% (denoted as M2 in FDA guidance document) means that a relative proportion of 80% of the standard treatment effect is preserved in treatment arms that are declared non-inferior on the primary endpoint. This exceeds the general recommendation in the FDA guidance document of preserving at least 50% of the treatment effect.

A 12% margin was used for a phase 3 regimen trial (the STAND trial) evaluating a novel regimen for the treatment of drug susceptible-TB that was initiated around the same time as this trial (Tweed et al, 2021).

The projected proportion of participants with unsatisfactory outcome of 10% was based on the folowing assumptions:

(i) the proportion with ongoing requirement for TB drug treatment or with ongoing TB disease activity at week 96 who will be classified as unsatisfactory outcome is estimated as 2%. This is based on data from REMoxTB trial (Gillespie et al, 2014) that shows that <1% of patients on the control arm initiated treatment in the last 26 weeks of follow-up (and were therefore on treatment at the end of follow-up) and <1% had positive cultures at the end of follow-up.

(ii) The proportion who will have died by week 96 (minus those who died after week 48 and for reasons clearly unrelated to TB or treatment – classified as un-assessable) is estimated as 3% based on 2.5% of deaths in REMoxTB in the control arm.

(iii) The proportion who fail to attend at week 96 and who cannot be contacted and confirmed to be clinically well is assumed to be 5%. This is difficult to estimate precisely from previous trials which were not designed as treatment strategy trials and so patients were not often followed for the full duration if retreatment was started, but it was predicted to be unlikely to exceed 5% in this trial.

References:
Tiemersma EW, van der Werf MJ, Borgdorff MW, Williams BG, Nagelkerke NJ. Natural history of tuberculosis: duration and fatality of untreated pulmonary tuberculosis in HIV negative patients: a systematic review. *PloS one* 2011; **6**(4): e17601

Non-Inferiority Clinical Trials to Establish Effectiveness; Guidance for Industry; U.S. Department of Health and Human Services, Food and Drug Administration; Center for Drug Evaluation and Research (CDER); Center for Biologics Evaluation and Research (CBER); November 2016
https://www.fda.gov/media/78504/download

Tweed CD, Wills GH, Crook AM et al. A partially randomised trial of pretomanid, moxifloxacin and pyrazinamide for pulmonary TB. Int J TB Lung Dis 2021: 25(4):305–314.

Gillespie SH, Crook AM, McHugh TD et al. Four-month moxifloxacin-based regimens for drug-sensitive tuberculosis. NEJM 2014; 371: 1588-98.

Section S12 ANALYSIS OF THE PRIMARY OUTCOME

The approach to the analysis of the primary outcome is described in the Statistical Analysis Plan, section 8.6.1. The decision algorithm allocated an outcome to all participants and hence there was no missing data on the primary outcome.

The final model used for analysis of the primary outcome (unsatisfactory outcome, yes or no) was a generalized linear model with binomial distribution and identity link function adjusting for treatment arm (TRUNCATE strategy [rifampicin-linezolid]; TRUNCATE strategy [bedaquiline-linezolid]; and standard treatment); country (India and Thailand combined; Philippines and Indonesia combined; and Uganda); and relapse risk (lower; intermediate and higher combined). Combining categories, as indicated, was done to achieve convergence of the model.

The model fit was assessed using Deviance, a goodness of fit statistic to compare statistical models. The [deviance/degrees of freedom] value for the primary model was 0.7585, which is regarded as a good fit (a value of 1 indicates perfect fit).

Section S13 ANALYSIS OF SECONDARY OUTCOMES AND APPROACH TO MISSING DATA

There was no missing data for treatment days, adherence and transmission risk. Missing data on acceptability scores was negligible (3% overall) and a complete-case analysis was performed.

Missing data on health status (EQ-5D), body weight and body mass index at week 96 were handled by the analysis using linear mixed effect models with repeated measurements, adjusted for treatment arm, country, relapse risk and baseline value of the outcome. Visit is considered as a repeat effect in the model. These models take care of missing data implicitly and produce unbiased estimates for treatment arms.

For spirometry measurements, missing values of FEV1 were handled by multiple imputation, based on observed values from participants within the same treatment arm using full conditional specification method with linear regression model. The imputation model included variables for age; sex; height; country; proportion of lung affected and presence or absence of cavitation on baseline chest radiograph; and FEV1 values at preceding timepoints. Missing values of FEV1 were imputed 20 times to generate 20 complete datasets. The presence or absence of respiratory disability at week 96 and new respiratory disability at week 96 was determined for each participant in each of the datasets, as described in Section S7. The proportion difference between arms and corresponding 95% confidence intervals for respiratory disability at week 96 were estimated for each dataset using a generalized linear model with binomial distribution and identity link function, adjusting for treatment arm and country. For change in FEV1, a simple arithmetic mean and SD was calculated for each dataset.

For quality of life (MOS-HIV questionnaire) assessments, missing values on the mental health and physical health summary scores at week 96 were imputed (separately) using a similar approach, as described above for FEV1. The imputation model included variables for age; sex; country; and baseline summary score. The adjusted mean difference between the standard treatment arm and each complete TRUNCATE strategy arm and its 95% confidence interval in each dataset were derived using a linear fixed effects model, adjusted for treatment arm, country, relapse risk, and baseline values of the summary score.

For MRC breathlessness scale assessments, missing values at week 96 were imputed using a similar approach as described for FEV1 but using a logistic regression model which included variables for age, sex country and values at previous timepoints. The proportion difference between arms and corresponding 95% confidence intervals for MRC breathlessness scale grade ≥ 3 at week 96 were estimated for each dataset using normal approximation to binomial distribution.

The estimates and confidence intervals obtained from each dataset were combined based on Rubin's formula (Rubin, 1987) to obtain the final estimate for the mean or proportion for each treatment arm (mean for change in FEV1 and proportion for respiratory disability on FEV1 < 50% and MRC breathlessness scale ≥ 3), mean or proportion difference between arms (mean for change in mental health and physical health summary scores and proportion for respiratory disability on FEV1 < 50% and MRC breathlessness scale ≥ 3) and their respective 95% confidence intervals.

Rubin, D.B. (1987) Multiple Imputation for Nonresponse in Surveys. John Wiley & Sons Inc., New York.

SUPPLEMENTARY TABLES

Table S1 Representativeness of study participants

Disease, problem or condition under investigation	Rifampicin-susceptible pulmonary tuberculosis
Considerations related to sex or gender	Tuberculosis affects men more than women; in 2021, 63% of adult cases globally occurred in men.[1]
Considerations related to age	Tuberculosis affects adults more than children. In 2021, 89% of tuberculosis cases globally occurred in adults, 11% in children. Amongst adults, most infections occur in the most productive years[1].
Considerations related to race or ethnic group	There are no biological associations between race or ethnicity and the risk of drug-susceptible TB.
Considerations related to geography	Most people who developed tuberculosis in 2021 were in the WHO regions of South-East Asia (45%), Africa (23%) and the Western Pacific (18%). Eight countries account for more than two thirds of the global total – these include India (28%), Indonesia (9.2%), and the Philippines (7.0%).[1]
Other considerations	Undernutrition, HIV infection, alcohol use disorders, smoking and diabetes are major risk factors for tuberculosis and are common co-morbidities in people with TB; 6.7% of incident cases of TB globally have HIV co-infection.[1] Tuberculosis disease severity indices vary by study selection criteria, but studies of population screening for tuberculosis show 10% - 79% of culture confirmed cases are smear positive and 3-33% of smear positive cases are of grade 3+).[2]
Overall representativeness of the trial	62% of the participants were male, representative of global gender distribution of tuberculosis.<br>All participants were adults of working age (18-65 years old, median age 32), representative of global adult age distribution of tuberculosis. No children were enrolled.<br>All trial participants were recruited from countries that are in the top 30 high-tuberculosis-burden countries; 82% of participants were recruited from countries in the top 8 high-tuberculosis-burden countries (India, Indonesia and Philippines). The trial recruited participants from both Asia and Africa and is broadly

	representative of global geographic and ethnic distribution, although there was no representation of participants from Europe or The Americas and those of white ethnicity.<br>At trial entry, 43% of trial participants had undernutrition (BMI <18.5 kg/m^2); 49% of participants reported current or prior smoking history; 9% of participants had diabetes, broadly representative of these major risk factors in the global population of people with tuberculosis.<br>The trial population comprised 31% participants who reported drinking alcohol (2% with heavy intake) but excluded those with known alcohol abuse. The trial may under-represent the proportion of people with tuberculosis globally with alcohol use disorders; at a programme level such people may benefit from the strategy with a shorter initial treatment period and increased resources given to adherence support but maintaining post-treatment monitoring may be more challenging.<br>The trial initially excluded those with HIV infection; enrolment of selected HIV co-infected participants was permitted in the last 2-3 months of enrolment at most sites, but none were enrolled. The trial is therefore not representative with respect to the 6.7% global prevalence of HIV co-infection in tuberculosis patients and the strategy will require additional evaluation in this group. However, participants on stable anti-retroviral therapy with high CD4 T-cell counts would be expected to do well.<br>The trial initially excluded participants with sputum smear grade 3+ or large (>4cm) cavities on chest radiograph at screening (but did not exclude participants based on radiographic extent of disease); enrolment of participants with these factors was permitted in the last 2-3 months of enrolment at most sites. Overall, the baseline disease burden in the trial population (73% smear positive; 18% of those smear positive had grade 3+; XpertMTB/RIF medium/high in 61%; 54% with cavitation on chest radiography) is representative of disease severity indices described in studies of population screening for tuberculosis.[2]

Reference(s):

1. Global Tuberculosis Report 2022 – TB Data Hub. Citation: Global tuberculosis report 2022. Geneva: World Health Organization; 2022 ISBN 978-92-4-006172-9

2. Telesinghe et al. Does tuberculosis screening improve individual outcomes? A systematic review. E Clinical Medicine 2021; 40 101127 (doi: 10.1016/j.eclinm.2021.101127)

Table S2 Initial treatment course completion and switches *

	Standard treatment (N= 181)	TRUNCATE strategy (hRIF/LZD) (N=184)	TRUNCATE strategy (hRIF/CFZ) (N=78)	TRUNCATE strategy (RPT/LZD) (N=42)	TRUNCATE strategy (BDQ/LZD) (N=189)	TRUNCATE strategy (all) (N=493)
**TRUNCATE-TB strategy arms**						
**Completed treatment with assigned regimen only (without extension with, or switch to, standard treatment) †**						
Overall – no (%)	-	169 (92)	72 (92)	31 (74)	179 (95)	451 (91)
≥ 54 to 56 qualifying days – no (%)	-	143 (78)	60 (77)	28 (67)	162 (86)	393 (80)
≥ 57 to 70 qualifying days – no (%) ‡	-	21 (11)	8 (10)	2 (5)	13 (7)	44 (9)
≥ 71 to 84 qualifying days - no (%) §	-	5 (3)	4 (5)	1 (2)	4 (2)	14 (3)
Total qualifying days – days	-	58.4 ± 6.1	58.6 ± 6.7	57.5 ± 5.6	57.7 ± 5.3	58.1 ± 5.8
**Completed treatment with assigned regimen for 84 qualifying days; extended and completed treatment with standard treatment †**						
Overall - no. (%) ¶	-	3 (2)	1 (1)	0	3 (2)	7 (1)
**Did not complete treatment with assigned regimen; switched and completed treatment with standard treatment †**						
Overall - no. (%)	-	7 (4)	4 (5)	9 (21)	5 (3)	25 (5)
Reason(s) for switch – no. ‖						
Missed doses **	-	1	1	2	0	4
Adverse events	-	4	4	8	3	16
Pill burden/tolerability	-	2	0	3	1	6
Decision by participant (other reason)	-	3	0	1	1	5
Other ††	-	0	0	0	1	1
**Did not complete treatment with assigned regimen; did not complete treatment with standard treatment †**						
Overall no. (%)	-	5 (3)	1 (1)	2 (5)	2 (1)	10 (2)
Died before completion of initial treatment – no.	-	1	0	0	0	1
Withdrew from trial follow-up – no.	-	1	0	0	0	1
Defaulted treatment, remained under follow-up – no.	-	3	1	2	2	8
**Standard treatment arm**						
Completed standard treatment- no. (%) †	178 (98)	-	-	-	-	-
Did not complete standard treatment - no. (%)	3 (2)	-	-	-	-	-
Died before completion of initial treatment – no.	2	-	-	-	-	-
Defaulted treatment, remained under follow-up – no.	1	-	-	-	-	-

*Plus-minus values are means ± SD. In the TRUNCATE strategy arms, hRIF/LZD denotes the initial treatment regimen including high-dose rifampicin and linezolid; hRIF/CFZ denotes the initial treatment regimen including high-dose rifampicin and clofazimine; RPT/LZD denotes the initial treatment regimen including rifapentine and linezolid; BDQ/LZD denotes the initial treatment regimen including bedaquiline and linezolid. Percentages may not total 100 because of rounding.

† Completed assigned regimen is defined as taking at least 54 qualifying days of the randomly-assigned regimen in the TRUNCATE strategy arms; completed standard treatment is defined as taking at least 154 qualifying days of the standard regimen only (or at least 154 total qualifying days of either the assigned regimen or standard treatment in the TRUNCATE strategy arms). A qualifying day is a day on which treatment was taken with at least 50% of the protocol-mandated dose of all allocated drugs in the regimen (exceptions and detailed definition in protocol and statistical analysis plan).

‡ Extension with the assigned regimen to complete treatment after a total of ≥57 to 70 days was for persistent clinical disease (symptoms and positive smear) in 12; symptoms with either negative smear or no sputum available in 25; positive smear without symptoms in 2; miscalculation of days in 4; and clinical decision in 1 participant.

§ Extension with the assigned regimen to complete treatment after a total of ≥71 to 84 days was for persistent clinical disease (symptoms and positive smear) in 9; and symptoms with either negative smear or no sputum available in 5 participants. One participant classified in this category took 85 qualifying days of the assigned regimen in error.

¶ Extension with standard treatment after a total of 84 days of the assigned regimen was for persistent clinical disease (symptoms and positive smear) in 6, and symptoms with either negative smear or no sputum available in 1 participant.

‖ Participants may have more than 1 reason for switch

** Switch to standard treatment was mandated for treatment interruption lasting more than 14 consecutive days or if the required number of days of the assigned initial regimen in a TRUNCATE strategy arm could not be completed by week 12.

†† Interruption of drug supply in one patient

Table S3 Re-treatment courses *

	Standard treatment (N= 181)	TRUNCATE strategy (hRIF/LZD) (N=184)	TRUNCATE strategy (hRIF/CFZ) (N=78)	TRUNCATE strategy (RPT/LZD) (N=42)	TRUNCATE strategy (BDQ/LZD) (N=189)		TRUNCATE strategy (all) (N=493)
Started second treatment course – no. (%)							
Total – no. (%) †	6 (3)	42 (23)	10 (13)	8 (19)	24 (13)		84 (17)
Completed first treatment course – no. ‡	5	39	9	8	24		80
Did not complete first treatment course - no.	1	3	1	0	0		4
Time to start of second treatment course – days							
From end of initial treatment course	274 ± 97	230 ± 160	229 ± 123	235 ± 123	277 ± 122		243 ± 142
From baseline day	424 ± 113	287 ± 158	289 ± 129	292 ± 124	340 ± 122		303 ± 142
Started third treatment course – no. (%)							
Total - no. (%) §	0	1 (1)	1 (1)	0	0		2 (<1)
Completed second treatment course – no. ¶	0	1	1	0	0		2
Did not complete second treatment course - no.	0	0	0	0	0		0

*Plus-minus values are means ± SD. In the TRUNCATE strategy arms, hRIF/LZD denotes the initial treatment regimen including high-dose rifampicin and linezolid; hRIF/CFZ denotes the initial treatment regimen including high-dose rifampicin and clofazimine; RPT/LZD denotes the initial treatment regimen including rifapentine and linezolid; BDQ/LZD denotes the initial treatment regimen including bedaquiline and linezolid.

†The second course of treatment comprised standard treatment alone in 69 (82%); standard treatment with pyrazinamide omitted in 2 (2%); standard treatment with levofloxacin added in 7 (8%); standard treatment with pyrazinamide omitted and levofloxacin added in 6 (7%).

† Completed first treatment course is defined taking at least 54 qualifying days of the randomly-assigned regimen in the TRUNCATE strategy arms in those who took only the assigned regimen; or taking at least 154 qualifying days of standard treatment in the standard management strategy arm; or taking at least 154 total qualifying days (of the assigned regimen and standard treatment combined) for those in the TRUNCATE strategy arms who switched from assigned regimen to standard treatment, or who extended with standard treatment following the completion of the assigned regimen. A qualifying day is a day on which the treatment was taken with at least 50% of the protocol-mandated dose of all allocated drugs in the regimen (exceptions and detailed definition in protocol and statistical analysis plan).

§ The third course of treatment comprised standard treatment in 1 participant and standard treatment with levofloxacin added in 1 participant

¶ Completed second treatment course is defined as taking at least 154 qualifying days of standard treatment in the second treatment course. Qualifying day defined as above.

Table S4 Primary efficacy outcome, proportions at week 96 in the intention to treat population, all arms *

Outcome	Standard treatment (N= 181)	TRUNCATE strategy (hRIF/LZD) (N=184)	TRUNCATE strategy (hRIF/CFZ) (N=78)	TRUNCATE strategy (RPT/LZD) (N=42)	TRUNCATE strategy (BDQ/LZD) (N=189)
**Participants with unsatisfactory outcome – no (%) †**	**7 (3.9)**	**21 (11.4)**	**8 (10.3)**	**2 (4.8)**	**11 (5.8)**
On tuberculosis treatment at week 96	2 (1.1)	8 (4.3)	2 (2.6)	1 (2.4)	5 (2.6)
Tuberculosis disease activity at week 96 – definitive §	1 (0.6)	4 (2.2)	6 (7.7)	0	3 (1.6)
Death before week 96 ¶	2 (1.1)	5 (2.7)	0	1 (2.4)	1 (0.5)
Telephone evaluation – well but insufficient evidence of disease clearance when last seen	2 (1.1)	3 (1.6)	0	0	1 (0.5)
No week 96 evaluation – insufficient evidence of disease clearance when last seen	0	1 (0.5)	0	0	1 (0.5)
**Participants with unassessable outcome – no (%)**	**1 (0.6)**	**1 (0.5)**	**0**	**0**	**2 (1.1)**
Single positive culture at week 96, no other evidence of disease activity ‖	0	1 (0.5)	0	0	0
Death from cause definitely unrelated to tuberculosis **	1 (0.6)	0	0	0	0
No week 96 evaluation – evidence of disease clearance when last seen	0	0	0	0	2 (0.9)
**Participants with satisfactory outcome – no (%)**	**173 (95.6)**	**162 (88.0)**	**70 (89.7)**	**40 (95.2)**	**176 (93.1)**

* In the TRUNCATE strategy arms, hRIF/LZD denotes the initial treatment regimen including high-dose rifampicin and linezolid; hRIF/CFZ denotes the initial treatment regimen including high-dose rifampicin and clofazimine; RPT/LZD denotes the initial treatment regimen including rifapentine and linezolid; BDQ/LZD denotes the initial treatment regimen including bedaquiline and linezolid.

† Unsatisfactory outcome, the primary outcome, was determined by a pre-specified algorithm (Section S10). Of the 660 participants alive and under follow up at week 96, 643 (97%) were evaluated in person, 17 (3%) by telephone; of those evaluated in person, 637 (99%) had a chest radiograph performed, 613 (95%) produced at least 1 evaluable sputum sample or were asymptomatic and were unable to produce sputum (imputed negative), and 28 (4%) produced sputum samples that were not evaluable.

§ Cases (14) with definitive disease activity at week 96 had at least two positive sputum cultures (13); or a positive sputum culture and positive smear on separate samples (1). Whole genome sequencing was available in paired isolates at week 96 and baseline (week 10 in one case where no baseline strain available) in 10 of the 14 cases; all were shown to be related to baseline strain, consistent with relapse. There were no cases of presumptive or possible disease activity at week 96.

¶ Causes of death are listed in the footnote to Table S11

‖ Whole genome sequencing showed the single positive culture at week 96 to be related to baseline strain.

** Cause of death was cervical cancer

Table S5 Primary efficacy outcome, proportions at week 96 in the assessable population, all arms

Outcome	Standard treatment (N= 180)	TRUNCATE strategy (hRIF/LZD) (N=183)	TRUNCATE strategy (hRIF/CFZ) (N=78)	TRUNCATE strategy (RPT/LZD) (N=42)	TRUNCATE strategy (BDQ/LZD) (N=187)
**Participants with unsatisfactory outcome – no (%)**	**7 (3.9)**	**21 (11.5)**	**8 (10.3)**	**2 (4.8)**	**11 (5.9)**
On tuberculosis treatment at week 96	2 (1.1)	8 (4.3)	2 (2.6)	1 (2.4)	5 (2.6)
Tuberculosis disease activity at week 96 - definitive	1 (0.6)	4 (2.2)	6 (7.7)	0	3 (1.6)
Death before week 96	2 (1.1)	5 (2.7)	0	1 (2.4)	1 (0.5)
Telephone evaluation – well but insufficient evidence of disease clearance when last seen	2 (1.1)	3 (1.6)	0	0	1 (0.5)
No week 96 evaluation – insufficient evidence of disease clearance when last seen	0	1 (0.5)	0	0	1 (0.5)
**Participants with satisfactory outcome – no (%)**	**173 (96.1)**	**162 (88.5)**	**70 (89.7)**	**40 (95.2)**	**176 (94.1)**

Table S6 Primary efficacy outcome, proportions at week 96 in the per-protocol population, all arms

Outcome	Standard treatment (N= 177)	TRUNCATE strategy (hRIF/LZD) (N=160)	TRUNCATE strategy (hRIF/CFZ) (N=70)	TRUNCATE strategy (RPT/LZD) (N=31)	TRUNCATE strategy (BDQ/LZD) (N=176)
**Participants with unsatisfactory outcome – no (%)**	**6 (3.4)**	**17 (10.6)**	**8 (11.4)**	**2 (6.4)**	**9 (5.1)**
On tuberculosis treatment at week 96	2 (1.1)	8 (5.0)	2 (2.9)	1 (3.2)	4 (2.3)
Tuberculosis disease activity at week 96 - Definitive	1 (0.6)	3 (1.9)	6 (8.6)	0	3 (1.7)
Death before week 96	2 (1.1)	4 (2.5)	0	1 (3.2)	0
Telephone evaluation – well but insufficient evidence of disease clearance when last seen	1 (0.6)	2 (1.3)	0	0	1 (0.6)
No week 96 evaluation – insufficient evidence of disease clearance when last seen	0	0	0	0	1 (0.6)
**Participants with unassessable outcome – no (%)**	**1 (0.6)**	**1 (0.6)**	**0**	**0**	**2 (1.1)**
Single positive culture at week 96, no other evidence of disease activity	0	1 (0.6)	0	0	0
Death from cause definitely unrelated to tuberculosis	1 (0.6)	0	0	0	0
No week 96 evaluation – evidence of disease clearance when last seen	0	0	0	0	2 (1.1)
**Participants with satisfactory outcome – no (%)**	**170 (96.0)**	**142 (88.8)**	**62 (88.6)**	**29 (93.5)**	**165 (93.8)**

Table S7 Primary efficacy outcome, difference in proportions at week 96 in the intention-to-treat population, all arms *

	Standard treatment	TRUNCATE strategy (hRIF/LZD)	TRUNCATE strategy (hRIF/CFZ)	TRUNCATE strategy (RPT/LZD)	TRUNCATE strategy (BDQ/LZD)
**Intention to treat population**					
Included in the population – no.	181	184	78	42	189
Unsatisfactory outcome – no.	7	21	8	2	11
Adjusted outcome †					
Proportion with unsatisfactory outcome (95% CI)	5.3 (1.5 to 9.0)	12.7 (7.4 to 18.0)	12.5 (3.5 to 21.6)	8.8 (-3.4 to 21.0)	6.1 (1.9 to 10.2)
Difference in proportion (95% CI)	N/A	7.4 (2.4 to 12.5)	8.6 (1.1 to 16.0)	1.9 (-6.4 to 10.3)	0.8 (-2.9 to 4.5)
Difference in proportion (97.5% CI)	N/A	7.4 (1.7 to 13.2)	8.6 (0.1 to 17.0)	1.9 (-7.6 to 11.5)	0.8 (-3.4 to 5.1)
Unadjusted outcome ‡					
Proportion with unsatisfactory outcome (95% CI)	3.9 (1.1 to 6.7)	11.4 (6.8 to 16.0)	10.3 (3.5 to 17.0)	4.8 (-1.7 to 11.2)	5.8 (2.5 to 9.2)
Difference in proportion (95% CI)	N/A	7.6 (2.2 to 13.0)	7.7 (0.05 to 15.3)	0.3 (-8.5 to 9.1)	1.9 (-2.4 to 6.3)
Difference in proportion (97.5% CI)	N/A	7.6 (1.4 to 13.7)	7.7 (-1.0 to 16.4)	0.3 (-9.8 to 10.4)	1.9 (-3.0 to 6.9)
Bayesian analysis §					
Proportion with unsatisfactory outcome (95% CI)	4.4 (1.9 to 7.7)	11.8 (7.7 to 16.8)	11.2 (5.3 to 19.1)	6.8 (1.5 to 15.6)	6.3 (3.3 to 10.0)
Difference in proportion (95% CI)	N/A	7.5 (2.1 to 13.0)	7.4 (-0.4 to 16.0)	0.4 (-10.0 to 10.8)	2.0 (-2.6 to 6.7)
Difference in proportion (97.5% CI)	N/A	7.5 (1.4 to 14.0)	7.4 (-1.7 to 17.5)	0.4 (-12.1 to 12.7)	2.0 (-3.2 to 7.3)
Probability of difference in proportion < 12%	N/A	0.944	0.871	0.983	1.000
Probability of difference in proportion < 6%	N/A	0.300	0.370	0.874	0.958

* Estimates of difference in proportions are presented with 97.5% confidence interval (or Bayesian credibility interval) to adjust for multiplicity; 95% confidence intervals are shown for information.

† Adjusted estimates shown for standard treatment and TRUNCATE strategy (rifampicin-linezolid) and (bedaquiline-linezolid) arms are from a generalized linear model with binomial distribution, with adjustment for country and relapse risk.

Adjusted estimates shown for the TRUNCATE strategy (rifampicin-clofazimine) arm are from a similar model adjusted for country only. The model included 77 participants in the standard treatment arm as contemporary randomised controls, in whom there occurred 2 unsatisfactory outcomes. The adjusted estimate for proportion with unsatisfactory outcome in the standard treatment arm in this model is 4.0 (-2.2 to 10.2) %.

Adjusted estimates shown for the TRUNCATE strategy (rifapentine-linezolid) arm are from a similar model adjusted for country only. The model included 45 participants in the standard treatment arm as contemporary randomised controls, in whom there occurred 2 unsatisfactory outcomes. The adjusted estimate for proportion with unsatisfactory outcome in the standard treatment arm in this model is 6.9 (-3.6 to 17.5) %.

‡ Unadjusted estimates shown for the TRUNCATE strategy (rifampicin-clofazimine) arm are based on 77 participants in the standard treatment arm as contemporary randomised controls, in whom there occurred 2 unsatisfactory outcomes (as above). The adjusted estimate for the proportion with unsatisfactory outcome in the standard treatment arm in this model is 2.6 (-0.96 to 6.1) %.

Unadjusted estimates shown for the TRUNCATE strategy (rifapentine-linezolid) arm are based on 45 participants in the standard treatment arm as contemporary randomised controls, in whom there occurred 2 unsatisfactory outcomes (as above). The adjusted estimate for the proportion with unsatisfactory outcome in the standard treatment arm in this model is 4.4 (-1.58 to 10.47) %.

§ Bayesian analysis used an unadjusted model with a flat uninformative prior distribution. Separate models were done for comparison of the TRUNCATE strategy (rifampicin-clofazimine) arm and TRUNCATE strategy (rifapentine-linezolid) arms individually with the standard treatment arm with appropriate contemporary controls (as for the other models above), with estimates for the proportion with unsatisfactory outcome in the standard treatment arm of 3.8% and 6.4% in the two models, respectively.

Table S8 Primary efficacy outcome, difference in proportions at week 96 in the assessable population, all arms *

	Standard treatment	TRUNCATE strategy (hRIF/LZD)	TRUNCATE strategy (hRIF/CFZ)	TRUNCATE strategy (RPT/LZD)	TRUNCATE strategy (BDQ/LZD)
**Assessable population**					
Included in the population – no.	180	183	78	42	188
Unsatisfactory outcome – no.	7	21	8	2	11
Adjusted outcome †					
Proportion with unsatisfactory outcome (95% CI)	5.4 (1.6 to 9.2)	12.8 (7.5 to 18.1)	12.5 (3.5 to 21.6)	8.8 (-3.4 to 21.0)	6.2 (2.0 to 10.4)
Risk difference (95% CI)	N/A	7.5 (2.4 to 12.5)	8.5 (1.1 to 15.9)	1.9 (-6.4 to 10.3)	0.8 (-2.9 to 4.6)
Risk difference (97.5% CI)	N/A	7.5 (1.7 to 13.2)	8.5 (0.1 to 17.0)	1.9 (-7.6 to 11.5)	0.8 (-3.4 to 5.1)

***** Estimates of difference in proportions are presented with 97.5% confidence interval to adjust for multiplicity; 95% confidence intervals are shown for information.

† Estimates shown for standard treatment and TRUNCATE strategy (rifampicin-linezolid) and (bedaquiline-linezolid) arms are from a generalized linear model with binomial distribution, with adjustment for country and relapse risk.

Estimates shown for the TRUNCATE strategy (rifampicin-clofazimine) arm are from a similar model adjusted for country only. The model included 77 participants in the standard treatment arm as contemporary randomised controls, in whom there occurred 2 unsatisfactory outcomes. The adjusted estimate for proportion with unsatisfactory outcome in the standard treatment arm in this model is 4.0 (-2.2 to 10.2) %.

Estimates shown for the TRUNCATE strategy (rifapentine-linezolid) arm are from a similar model adjusted for country only. The model included 45 participants in the standard treatment arm as contemporary randomised controls, in whom there occurred 2 unsatisfactory outcomes. The adjusted estimate for proportion with unsatisfactory outcome in the standard treatment arm in this model is 6.9 (-3.6 to 17.5) %.

Table S9 Primary efficacy outcome, difference in proportions in the per-protocol population, all arms *

	Standard treatment	TRUNCATE strategy (hRIF/LZD)	TRUNCATE strategy (hRIF/CFZ) †	TRUNCATE strategy (RPT/LZD) ‡	TRUNCATE strategy (BDQ/LZD)
**Per-protocol population**					
Included in the population – no.	177	160	70	31	176
Unsatisfactory outcome – no.	6	17	8	2	9
Outcome (adjusted or unadjusted) †					
Proportion with unsatisfactory outcome (95% CI)	4.9 (1.2 to 8.5)	11.7 (6.3 to 17.1)	11.4 (4.0 to 18.9)	6.4 (-2.2 to 15.1)	5.8 (1.7 to 9.9)
Risk difference (95% CI)	N/A	6.9 (1.7 to 12.1)	10.1 (2.2 to 18.0)	4.2 (-5.5 to 13.9)	0.9 (-2.8 to 4.6)
Risk difference (97.5% CI)	N/A	6.9 (0.9 to 12.8)	10.1 (1.1 to 19.1)	4.2 (-7.0 to 15.3)	0.9 (-3.3 to 5.1)

* Estimates of difference in proportions are presented with 97.5% confidence interval to adjust for multiplicity; 95% confidence intervals are shown for information.

† Estimates shown for standard treatment and TRUNCATE strategy (rifampicin-linezolid) and (bedaquiline-linezolid) arms are from a generalized linear model with binomial distribution, with adjustment for country and relapse risk. The 97.5% confidence interval is shown to adjust for multiplicity.

Estimates shown for the TRUNCATE strategy (rifampicin-clofazimine) arm are from an unadjusted model. The model included 75 participants in the standard treatment arm as contemporary randomised controls, in whom there occurred 1 unsatisfactory outcome. The estimate for proportion with unsatisfactory outcome in the standard treatment arm in this model is 1.3 (-1.3 to 3.9) %.

Estimates shown for the TRUNCATE strategy (rifapentine-linezolid) arm are from an unadjusted model. The model included 44 participants in the standard treatment arm as contemporary randomised controls, in whom there occurred 1 unsatisfactory outcome. The estimate for proportion with unsatisfactory outcome in the standard treatment arm in this model is 2.3 (-2.1 to 6.7) %.

Table S10 Acceptability, all arms *

	Standard treatment (N= 181)	TRUNCATE strategy (hRIF/LZD) (N=184)	TRUNCATE Strategy (hRIF/CFZ) (N=78)	TRUNCATE strategy (RPT/LZD) (N=42)	TRUNCATE strategy (BDQ/LZD) (N=189)
**Difficulty**					
Difficulty score	1.5 ± 1.7	2.4 ± 2.2	2.1 ± 1.0	2.6 ± 1.0	1.8 ± 2.0
Acceptable on difficulty domain, overall - no (%)	159 /177 (90)	139/176 (79)	65/78 (83)	33/42 (79)	158/180 (88)
Acceptable, swallowing pills – no (%)	165/177 (93)	150/176 (85)	67/78 (86)	33/42 (79)	162/180 (90)
Acceptable, post-treatment visits - no (%)	169/177 (95)	157/176 (89)	75/78 (96)	42/42 (100)	170/180 (94)
**Anxiety**					
Anxiety score	3.6 ± 2.2	3.9 ± 2.0	3.8 ± 1.9	3.9 ± 2.0	3.4 ± 2.0
Acceptable on anxiety domain - no (%)	117/177 (66)	109/176 (62)	45/78 (58)	25/42 (60)	119/180 (66)
Acceptable, risk of side effects – no (%)	156/177 (88)	157/176 (89)	72/78 (92)	38/42 (90)	160/180 (89)
Acceptable, risk of TB recurrence - no. (%)	147/177 (83)	148/176 (84)	64/78 (82)	38/42 (90)	149/180 (83)
Acceptable, risk of infecting others – no (%)	134/124 (76)	124/176 (70)	52/78 (67)	27/42 (64)	134/180 (74)
**Motivation**					
Motivation score	6.2 ± 3.9	8.0 ± 3.0	8.4 ± 2.4	8.2 ± 2.6	8.1 ± 2.9
Strategy increased motivation “a lot”- no. (%)					
None – no. (%)	38 (21.0)	10 (5.4)	1 (1.3)	1 (2.4)	7 (3.7)
A little – no. (%)	22 (12.2)	18 (9.8)	7 (9.0)	4 (9.5)	25 (13.2)
Some – no. (%)	44 (24.3)	40 (21.7)	19 (24.4)	11 (26.2)	33 (17.5)
A lot – no. (%)	73 (40.3)	108 (58.7)	51 (65.4)	26 (61.9)	115 (60.8)
**Recommendation to others**					
2-month treatment – no. (%)	NA	126 (68.5)	56 (71.8)	31 (73.8)	141 (74.6)
6-month treatment – no. (%)	NA	35 (19.0)	9 (11.5)	6 (14.3)	25 (13.2)
No preference – no. (%)	NA	15 (8.2)	13 (16.7)	5 (11.9)	14 (7.4)

* Plus-minus values are means ± SD.
Acceptability scores were derived from participant responses to questions on difficulty (swallowing pills, attending post-treatment clinic visits) or anxiety (risk of side effects, recurrence of tuberculosis or infecting others), assessed at week 48 and 96; and to a question on how much the planned treatment duration increased adherence motivation when starting treatment, based on the response at week 48; responses were normalized to a scale of 10 (0, “none”; 10, “a lot”). Overall acceptability of the strategy was assessed from the response at week 96 to a question about which treatment option the participant would recommend to a friend (evaluated in the TRUNCATE strategy arms only). Details of derivation in Section 9 and in the statistical analysis plan.

Table S11 Secondary outcomes, all arms

	Standard treatment<br><br>(N= 181)	TRUNCATE strategy (hRIF/LZD) (N=184)	TRUNCATE strategy (hRIF/CFZ) (N=78)	TRUNCATE strategy (RPT/LZD) (N=42)	TRUNCATE strategy (BDQ/LZD) (N=189)
**Participant-centred outcomes**					
Total treatment days to week 96					
Total duration of treatment courses – days †	180.2 ± 37.9	105.7 ± 80.1	98.7 ± 98.2	123.3 ± 79.9	84.8 ± 65.3
Total qualifying days of treatment – days †	177.3 ± 35.6	101.6 ± 74.9	94.7 ± 91.8	117.2 ± 74.6	83.8 ± 64.2
Acceptability ‡					
Difficulty score	1.5 ± 1.7	2.4 ± 2.2	2.1 ± 1.0	2.6 ± 1.0	1.8 ± 2.0
Anxiety score	3.6 ± 2.2	3.9 ± 2.0	3.8 ± 1.9	3.9 ± 2.0	3.4 ± 2.0
Motivation score	6.2 ± 3.9	8.0 ± 3.0	8.4 ± 2.4	8.2 ± 2.6	8.1 ± 2.9
Recommendation to others					
2-month treatment – no. (%)	NA	126 (68.5)	56 (71.8)	31 (73.8)	141 (74.6)
6-month treatment – no. (%)	NA	35 (19.0)	9 (11.5)	6 (14.3)	25 (13.2)
No preference – no. (%)	NA	15 (8.2)	13 (16.7)	5 (11.9)	14 (7.4)
Quality of life (MOS-HIV questionnaire) §					
Mental health summary score	57.5 ± 0.5	57.5 ± 0.5	56.2 ± 0.8	57.6 ± 0.8	57.8 ± 0.5
Physical health summary score	56.7 ± 0.5	56.8 ± 0.5	56.8 ± 0.8	57.5 ± 0.8	56.7 ± 0.5
Health status (EQ-5D index score) ¶	0.99 ± 0.0	0.98 ± 0.1	0.98 ± 0.1	0.99 ± 0.0	0.98 ± 0.1
Illness-related missed work or study – days	2.6 ± 9.1	3.3 ± 9.4	4.6 ± 14.3	5.5 ± 12.6	3.1 ± 12.9
Body weight ‖					
Change from baseline – kg	5.8 ± 4.8	5.6 ± 4.7	6.6 ± 5.6	7.6 ± 5.6	6.1 ± 4.8
Change from baseline - %	11.9 ± 10.0	11.4 ± 9.8	13.5 ± 11.7	15.5 ± 11.2	12.1 ± 9.8
Body mass index					
Change from baseline - kg/m^2	2.3 ± 1.8	2.2 ± 1.9	2.5 ± 2.0	2.9 ± 2.1	2.3 ± 1.9
**Safety outcomes**					
Adverse events to week 96					
Participants with any grade 3 or 4 adverse event – no. (%)	29 (16.0)	32 (17.4)	12 (15.4)	11 (26.2)	30 (15.9)
Participants with any serious adverse event – no. (%)	11 (6.1)	18 (9.8)	10 (12.8)	4 (9.5)	14 (7.4)
Death **	3 (1.7)	5 (2.7)	0	1 (2.4)	1 (0.5)

Respiratory disability at week 96					
MRC breathlessness scale ≥ 3 – no (%)††	0	2.7 (1.5)	0	0	2.7 (1.4)
FEV1% < 50% – no. (%) ‡‡	24.3 (13.4)	20.5 (11.1)	11.0 (14.2)	6.3 (15.0)	22.4 (11.8)
FEV1% < 50%, new – no. (%) ‡‡	7.2 (4.0)	6.7 (3.6)	2.8 (3.7)	2.1 (5.1)	6.9 (3.6)
FEV1 change to week 96 - L ‡‡	0.25 (0.05)	0.23 (0.05)	0.06 (0.08)	0.00 (0.11)	0.27 (0.06)
**Programme-centred outcomes**					
Treatment adherence §§					
Adherence over first 56 days - %	98.7± 4.9	96.9 ± 7.7	97.1 ± 9.4	96.6 ± 8.7	98.9 ± 3.2
Missing ≥14 doses in first 56 days – no. (%)	4 (2.2)	7 (3.8)	6 (7.7)	4 (9.5)	2 (1.1)
Default within first 56 days – no. (%)	1 (0.6)	3 (1.6)	1 (1.3)	0	1 (0.6)
Adherence over all treatment courses - %	98.7 ± 4.8	96.8 ± 7.6	97.3 ± 7.8	96.1 ± 8.9	99.0 ± 3.0
Acquired drug resistance – no. (%) ¶¶	0	0	0	0	1 (0.6)
Relapse-associated transmission risk ‖‖					
Transmission risk period – days	0.5 ± 4.3	2.6 ± 8.4	2.7 ± 15.4	1.7 ± 6.3	3.2 ± 14.1
New exposed household contacts – n	0.01 ± 0.15	0.01 ± 0.10	0.01 ± 0.11	0.0 ± 0.0	0.06 ± 0.4

* In TRUNCATE strategy arms, (hRIF/LZD) denotes the arm with initial treatment regimen including high-dose rifampicin and linezolid; hRIF/CFZ denotes the initial treatment regimen including high-dose rifampicin and clofazimine; RPT/LZD denotes the initial treatment regimen including rifapentine and linezolid; (BDQ/LZD) denotes the arm with initial treatment regimen including bedaquiline and linezolid. MOS-HIV questionnaire denotes the Medical Outcomes Study-HIV questionnaire. MRC denotes Medical Research Council. Plus-minus values are means ± SD (mean ± SE for MRC breathlessness scale ≥ 3 and FEV1 change to week 96). The approach to missing data on secondary outcomes is described in Section S13.

† Total duration of treatment courses is the total time between the first and last day of each treatment course, summed for all courses from baseline to week 96 in each participant. Total qualifying days of treatment is the total number of days from baseline to week 96 on which the treatment was taken with at least 50% of the protocol-mandated dose of all allocated drugs in the regimen prescribed at that time (definitions in protocol and statistical analysis plan).

‡ Acceptability scores derived from a study-specific questionnaire (details in Section S9; additional data in Table S10). Acceptability scores were not available for 21 (3%) participants.

§ Quality of life scores were not available for 52 (8%) participants.

¶ Health status scores were not available for 38 (6%) participants

‖ Body weight and body mass index were not available for 40 (6%) participants

** Causes of death in the standard treatment arm were cervical carcinoma, cardiac arrest and possible cerebrovascular accident (each in 1 participant); in the TRUNCATE strategy (rifampicin-linezolid) arm were drug induced liver injury, COVID-19, and cirrhosis (each in 1 participant), and unknown cause (in 2 participants); in the TRUNCATE strategy (rifapentine-linezolid) arm was possible cerebrovascular accident; in the TRUNCATE strategy (bedaquiline-linezolid) arm was tuberculosis.

†† MRC breathlessness scale scores were not available for 48 (7%) participants

‡‡ FEV1%, measured by spirometry, is the maximum forced expiratory volume in 1 second, expressed as a percentage of the predicted value for an individual of the same age, sex, height and race (details in Section S7). FEV1 (and FEV1%) measurements were not available for 123 (18%) participants at week 8 and 87 (13%) participants at week 96; FEV1 change to week 96 was not available for 105 (16%) participants; missing values were imputed (Section S13). The number (%) of participants with FEV1% < 50% and FEV1% <50%, new (i.e. where FEV1% was ≥50% at week 8) are mean values obtained from multiple imputation.

§§ Adherence determined from number of qualifying days (defined above) over the first 56 days, or over the total period of all treatment courses; missed day refers to not taking treatment at a dose that counts as a qualifying day.

¶¶ Two participants in the TRUNCATE strategy (bedaquiline-linezolid) arm had confirmed acquired phenotypic drug resistance to bedaquiline (and clofazimine) accompanying relapse (weeks 36 and 52); both had 198 deletion in mmpR5 gene detected on whole genome sequencing. Results from additional 3 participants with unconfirmed resistance in the TRUNCATE strategy (rifampicin-linezolid) arm are listed in Table S16. Results based on phenotypic susceptibility obtained for 100% of disease recurrence episodes (at least one isolate tested to all exposed drugs in 873%; to all standard drugs in a further 9%; and to rifampicin, isoniazid and ethambutol only in a further 4%).

†† Transmission risk period is the time between the first smear of grade ≥ 1+ occurring during a confirmed relapse episode, and the time that re-treatment is started, or study follow-up ends. New exposed household contacts are those living in the household during the transmission risk period who were not doing so at trial entry.

Table S12 Grade 3 and 4 and Serious Adverse Events between baseline and week 96, all arms

	Standard treatment (N= 181)	TRUNCATE strategy (hRIF/LZD) (N=184)	TRUNCATE strategy (hRIF/CFZ) (N=78)	TRUNCATE strategy (RPT/LZD) (N=42)	TRUNCATE strategy (BDQ/LZD) (N=189)	Total (N=674)
Participants with adverse event of Grade 3 or 4 severity:						
Any event – no. (%)	29 (16.0)	32 (17.4)	12 (15.4)	11 (26.2)	30 (15.9)	114 (16.9)
Event related to TB medication – no. (%) *	13 (7.2)	13 (7.1)	6 (7.7)	8 (19.0)	18 (9.5)	58 (8.6)
Event related to TB disease – no. (%) †	5 (2.8)	9 (4.9)	2 (2.6)	4 (9.5)	6 (3.2)	26 (3.9)
Event related to relapse or retreatment – no (%) ‡	1 (0.6)	5 (2.7)	1 (1.3)	1 (2.4)	1 (0.5)	9 (1.3)
Participants with serious adverse event:						
Any event – no. (%)	11 (6.1)	18 (9.8)	10 (12.8)	4 (9.5)	14 (7.4)	57 (8.5)
Event related to TB medication – no. (%)	3 (1.7)	6 (3.3)	4 (5.1)	3 (7.1)	3 (1.6)	19 (2.8)
Event related to TB disease – no. (%)	2 (1.1)	8 (4.3)	2 (2.6)	0	3 (1.6)	15 (2.2)

* Event related to TB medication means event assessed by site investigator as at least possibly related to one or more study medications

† Event related to TB disease means event assessed by site investigator as at least possibly related to TB disease

‡ Event related to relapse or retreatment defined as event occurring from 4 weeks prior to treatment failure or relapse (confirmed or suspected) until the completion of re-treatment or the week 96 visit (whichever occurs first); and considered by the site investigator to be related to TB medication or TB disease (defined above). In the standard treatment arm the event was community acquired pneumonia, accompanying possible TB relapse (not culture confirmed), considered related to TB disease; in the TRUNCATE strategy (rifampicin-linezolid) arm events were cough at time of confirmed relapse, considered related to TB disease; community acquired pneumonia with lung abscess, accompanying possible TB relapse (not culture confirmed), considered related to TB disease; arthralgia at week 4 of retreatment course, considered related to treatment; sudden death (unknown cause) following confirmed treatment failure prior to restarting treatment, considered related to disease; death (unknown cause), 26 weeks following confirmed relapse in participant who refused re-treatment and discontinued study visits (each in 1 participant); in the TRUNCATE strategy (rifampicin-clofazimine arm) the event was anaemia at time of confirmed relapse, considered related to TB disease; in the TRUNCATE strategy (rifapentine-linezolid) arm the event was weight loss at time of confirmed relapse, considered related to TB disease; in the TRUNCATE strategy (bedaquiline-linezolid) arm the event was hepatotoxicity at time of starting retreatment, considered related to TB medication.

Table S13 Grade 3 and 4 adverse events between baseline and week 96 by system organ class, all arms *

System Organ Class<br>Preferred Term	Standard treatment<br>(N= 181)	TRUNCATE strategy (hRIF/LZD)<br>(N=184)	TRUNCATE strategy (hRIF/CFZ)<br>(N=78)	TRUNCATE strategy (RPT/LZD)<br>(N=42)	TRUNCATE strategy (BDQ/LZD)<br>(N=189)	Total<br>(N=674)
Participants with at least one grade 3 or 4 AE – no (%)	29 (16.0)	32 (17.4)	12 (15.4)	11 (26.2)	30 (15.9)	114 (16.9)
Blood and lymphatic system disorders – no (%)	9 (5.0)	4 (2.2)	1 (1.3)	3 (7.1)	15 (7.9)	32 (4.7)
Anaemia	6 (3.3)	2 (1.1)	1 (1.3)	2 (4.8)	14 (7.4)	25 (3.7)
Neutropenia	3 (1.7)	1 (0.5)	0	0	1 (0.5)	5 (0.7)
Lymphadenopathy	0	0	0	1 (2.4)	0	1 (0.1)
Pancytopenia	0	1 (0.5)	0	0	0	1 (0.1)
Hepatobiliary disorders – no (%)	6 (3.3)	8 (4.3)	4 (5.1)	4 (9.5)	3 (1.6)	25 (3.7)
Hyperbilirubinaemia	1 (0.6)	4 (2.2)	1 (1.3)	3 (7.1)	0	9 (1.3)
Drug-induced liver injury	3 (1.7)	1 (0.5)	1 (1.3)	0	0	5 (0.7)
Hepatotoxicity	1 (0.6)	1 (0.5)	0	1 (2.4)	2 (1.1)	5 (0.7)
Hepatitis	1 (0.6)	0	2 (2.6)	0	0	3 (0.4)
Acute hepatic failure	0	1 (0.5)	0	0	0	1 (0.1)
Cholecystitis acute	0	0	0	0	1 (0.5)	1 (0.1)
Hepatic cirrhosis	0	1 (0.5)	0	0	0	1 (0.1)
Jaundice	0	0	1 (1.3)	0	0	1 (0.1)
Metabolism and nutrition disorders – no (%)	1 (0.6)	6 (3.3)	1 (1.3)	1 (2.4)	3 (1.6)	12 (1.8)
Decreased appetite	0	3 (1.6)	0	0	0	3 (0.4)
Hyperglycaemia	0	1 (0.5)	1 (1.3)	0	1 (0.5)	3 (0.4)
Diabetes mellitus	1 (0.6)	1 (0.5)	0	0	0	2 (0.3)
Hyponatraemia	0	1 (0.5)	0	0	0	1 (0.1)
Abnormal loss of weight	0	0	0	1 (2.4)	0	1 (0.1)
Diabetic ketoacidosis	0	0	0	0	1 (0.5)	1 (0.1)

System Organ Class<br>Preferred Term	Standard treatment<br>(N= 181)	TRUNCATE strategy (hRIF/LZD)<br>(N=184)	TRUNCATE strategy (hRIF/CFZ)<br>(N=78)	TRUNCATE strategy (RPT/LZD)<br>(N=42)	TRUNCATE strategy (BDQ/LZD)<br>(N=189)	Total<br>(N=674)
Gout	1 (0.6)	0	0	0	0	1 (0.1)
Hyperuricaemia	0	0	0	0	1 (0.5)	1 (0.1)
Hypokalaemia	1 (0.6)	0	0	0	0	1 (0.1)
Infections and infestations	2 (1.1)	4 (2.2)	2 (2.6)	0	4 (2.1)	12 (1.8)
Corona virus infection	1 (0.6)	1 (0.5)	0	0	0	2 (0.3)
Pneumonia	1 (0.6)	0	1 (1.3)	0	2 (1.1)	4 (0.6)
Pulmonary tuberculosis	0	0	0	0	1 (0.5)	1 (0.1)
Appendicitis	0	0	0	0	1 (0.5)	1 (0.1)
Appendicitis perforated	0	0	0	0	1 (0.5)	1 (0.1)
Gastroenteritis	0	0	1 (1.3)	0	0	1 (0.1)
Lung abscess	0	1 (0.5)	0	0	0	1 (0.1)
Tooth abscess	0	1 (0.5)	0	0	0	1 (0.1)
Bartholin's abscess	0	1 (0.5)	0	0	0	1 (0.1)
Gastrointestinal disorders	1 (0.6)	3 (1.6)	1 (1.3)	5 (11.9)	2 (1.1)	12 (1.8)
Vomiting	1 (0.6)	2 (1.1)	1 (1.3)	3 (7.1)	1 (0.5)	8 (1.2)
Nausea	0	0	0	2 (4.8)	0	2 (0.3)
Diarrhoea	0	0	0	0	1 (0.5)	1 (0.1)
Oesophageal varices haemorrhage	0	1 (0.5)	0	0	0	1 (0.1)
Investigations	4 (2.2)	2 (1.1)	1 (1.3)	0	4 (2.1)	11(1.6)
Electrocardiogram QTc prolonged	0	0	0	0	1 (0.5)	1 (0.1)
Alanine aminotransferase increased	3 (1.7)	0	0	0	1 (0.5)	4 (0.6)
Aspartate aminotransferase increased	0	2 (1.1)	0	0	0	2 (0.3)
Blood creatinine increased	0	0	0	0	1 (0.5)	1 (0.1)
Blood glucose increased	1 (0.6)	0	0	0	0	1 (0.1)
Blood potassium increased	0	0	0	0	1 (0.5)	1 (0.1)

System Organ Class<br>Preferred Term	Standard treatment<br>(N= 181)	TRUNCATE strategy (hRIF/LZD)<br>(N=184)	TRUNCATE strategy (hRIF/CFZ)<br>(N=78)	TRUNCATE strategy (RPT/LZD)<br>(N=42)	TRUNCATE strategy (BDQ/LZD)<br>(N=189)	Total<br>(N=674)
Weight decreased	0	0	1 (1.3)	0	0	1 (0.1)
Nervous system disorders	1 (0.6)	2 (1.1)	2 (2.6)	2 (4.8)	2 (1.1)	9 (1.3)
Cerebrovascular accident	1 (0.6)	1 (0.5)	1 (1.3)	1 (2.4)	0	4 (0.6)
Headache	0	0	0	1 (2.4)	1 (0.5)	2 (0.3)
Basal ganglia stroke	0	0	0	0	1 (0.5)	1 (0.1)
Dizziness	0	1 (0.5)	1 (1.3)	0	0	2 (0.3)
Respiratory, thoracic and mediastinal disorders	1 (0.6)	3 (1.6)	0	0	1 (0.5)	5 (0.7)
Pneumothorax	0	1 (0.5)	0	0	1 (0.5)	2 (0.3)
Asthma	0	1 (0.5)	0	0	0	1 (0.1)
Bronchospasm	1 (0.6)	0	0	0	0	1 (0.1)
Cough	0	1 (0.5)	0	0	0	1 (0.1)
General disorders and administration site conditions	1 (0.6)	3 (1.6)	1 (1.3)	0	0	5 (0.7)
Paradoxical drug reaction	0	0	1 (1.3)	0	0	1 (0.1)
Adverse drug reaction	0	1 (0.5)	0	0	0	1 (0.1)
Asthenia	1 (0.6)	0	0	0	0	1 (0.1)
Death (uncertain cause)	0	2 (1.1)	0	0	0	2 (0.3)
Vascular disorders	1 (0.6)	2 (1.1)	0	0	0	3 (0.4)
Hypotension	1 (0.6)	1 (0.5)	0	0	0	2 (0.3)
Hypertension	0	1 (0.5)	0	0	0	1 (0.1)
Musculoskeletal and connective tissue disorders	1 (0.6)	1 (0.5)	0	0	0	2 (0.3)
Arthralgia	0	1 (0.5)	0	0	0	1 (0.1)
Musculoskeletal pain	1 (0.6)	0	0	0	0	1 (0.1)

System Organ Class<br>Preferred Term	Standard treatment<br>(N= 181)	TRUNCATE strategy (hRIF/LZD)<br>(N=184)	TRUNCATE strategy (hRIF/CFZ)<br>(N=78)	TRUNCATE strategy (RPT/LZD)<br>(N=42)	TRUNCATE strategy (BDQ/LZD)<br>(N=189)	Total<br>(N=674)
Pregnancy, puerperium and perinatal conditions	0	0	0	0	1 (0.5)	1 (0.1)
Cephalo-pelvic disproportion	0	0	0	0	1 (0.5)	1 (0.1)
Hyperemesis gravidarum	0	0	0	0	1 (0.5)	1 (0.1)
Cardiac disorders	2 (1.1)	0	0	0	0	2 (0.3)
Cardiac arrest	1 (0.6)	0	0	0	0	1 (0.1)
Cardiac failure acute	1 (0.6)	0	0	0	0	1 (0.1)
Immune system disorders	0	1 (0.5)	0	0	0	1 (0.1)
Anaphylactic reaction	0	1 (0.5)	0	0	0	1 (0.1)
Injury, poisoning and procedural complications	0	0	0	0	1 (0.5)	1 (0.1)
Road traffic accident	0	0	0	0	1 (0.5)	1 (0.1)
Neoplasms benign, malignant and unspecified	1 (0.6)	0	0	0	0	1 (0.1)
Cervix cancer metastatic	1 (0.6)	0	0	0	0	1 (0.1)
Psychiatric disorders	0	0	0	0	1 (0.5)	1 (0.1)
Insomnia	0	0	0	0	1 (0.5)	1 (0.1)
Renal and urinary disorders	0	1 (0.5)	0	0	0	1 (0.1)
Acute kidney injury	0	1 (0.5)	0	0	0	1 (0.1)
Skin and subcutaneous tissue disorders	0	1 (0.5)	0	0	0	1 (0.1)
Rash	0	1 (0.5)	0	0	0	1 (0.1)

* Adverse event grading based on Division of AIDS Toxicity Criteria. QTc prolongation required QTcF ≥ 500ms. Coded according to Medical Dictionary for Regulatory Activities (MedDRA)

Table S14 Serious adverse events between baseline and week 96 by category, all arms

	Standard treatment (N= 181)	TRUNCATE strategy (hRIF/LZD) (N=184)	TRUNCATE strategy (hRIF/CFZ) (N=78)	TRUNCATE strategy (RPT/LZD) (N=42)	TRUNCATE strategy (BDQ/LZD) (N=189)	Total (N=674)
Patients with at-least one SAE, n (%)	11 (6.1)	18 (9.8)	10 (12.8)	4 (9.5)	14 (7.4)	57 (8.5)
Total SAEs, N	11	19	10	5	16	61
Fatal	3	5	0	1	1	10
Life-threatening	0	1	0	1	5	7
Resulting in prolonged hospitalisation	8	14	8	4	16	50
Persistent or major disability or incapacity	1	1	1	0	1	4
Congenital anomaly or birth defect	0	0	0	0	0	0
Other important medical condition	1	1	1	0	1	4

The number of categories for SAEs may exceed the total number of SAEs because each event may be classified into more than one category.

Table S15 Grade 3 and 4 adverse events between baseline and week 96 by system organ class, for standard treatment arm and TRUNCATE strategy (rifampicin-linezolid) arm, shown by period before and after rifampicin dose change

	Standard treatment All (N=181)	Standard treatment randomised before dose change* (n=91)	Standard treatment randomised after dose change* (N=90)	TRUNCATE strategy (hRIF/LZD) All (N=184)	TRUNCATE strategy (hRIF/LZD) randomised before dose change* (N=88)	TRUNCATE strategy (hRIF/LZD) randomised after dose change* (N=96)
Participant with at least one grade 3 or 4 adverse event – no (%)	29 (16.0)	12 (13.2)	17 (18.9)	32 (17.4)	17 (19.3)	15 (15.6)
Blood and lymphatic system disorders – no (%)	9 (5.0)	2 (2.2)	7 (7.8)	4 (2.2)	2 (2.3)	2 (2.1)
Hepatobiliary disorders – no (%)	6 (3.3)	4 (4.4)	2 (2.2)	8 (4.3)	6 (6.8)	2 (2.1)
Metabolism and nutrition disorders – no (%)	1 (0.6)	1 (1.1)	0	6 (3.3)	5 (5.7)	1 (1.0)
Infections and infestations – no (%)	2 (1.1)	1 (1.1)	1 (1.1)	4 (2.2)	1 (1.1)	3 (3.1)
Gastrointestinal disorders – no (%)	1 (0.6)	1 (1.1)	0	3 (1.6)	2 (2.3)	1 (1.0)
Investigations – no (%)	4 (2.2)	1 (1.1)	3 (3.3)	2 (1.1)	0	2 (2.1)
Nervous system disorders – no (%)	1 (0.6)	0	1 (1.1)	2 (1.1)	2 (2.3)	0
Respiratory, thoracic and mediastinal disorders – no (%)	1 (0.6)	0	1 (1.1)	3 (1.6)	0	3 (3.1)
General disorders and administration site conditions – no (%)	1 (0.6)	1 (1.1)	0	3 (1.6)	0	3 (3.1)
Vascular disorders – no (%)	1 (0.6)	1 (1.1)	0	2 (1.1)	2 (2.3)	0
Musculoskeletal and connective tissue disorders – no (%)	1 (0.5)	1 (1.1)	0	1 (0.5)	1 (1.1)	0
Cardiac disorders – no (%)	2 (1.1)	0	2 (2.2)	0	0	0
Immune system disorders – no (%)	0	0	0	1 (0.5)	1 (1.1)	0
Neoplasms, benign, malignant and unspecified – no (%)	1 (0.6)	0	1 (1.1)	0	0	0
Renal and urinary disorders - no (%)	0	0	0	1 (0.5)	0	1 (1.0)
Skin and subcutaneous tissue disorders – no (%)	0	0	0	1 (0.5)	0	1 (1.0)

Adverse event grading based on Division of AIDS Toxicity Criteria. Coded according to Medical Dictionary for Regulatory Activities (MedDRA). TRUNCATE strategy (hRIF/LZD) denotes the strategy arm with initial treatment regimen including high-dose rifampicin and linezolid.

* Participants in the TRUNCATE strategy (hRIF/LZD) arm who were randomised before the date of rifampicin dose change (1st November 2019) were prescribed a starting dose of 35mg/kg rifampicin. Participants in this arm randomised on or after the date of rifampicin dose change were prescribed a starting dose of dose of 20mg/kg rifampicin. Participants in the standard treatment arm were prescribed a starting dose of rifampicin 10mg/kg in both periods of randomisation; they are shown divided by date of randomisation to allow comparison of events in contemporaneously-randomised participants on standard treatment.

Table S16 Unconfirmed acquired drug resistance

**Treatment arm**	**Drug resistance (week first detected)**	**Details**
TRUNCATE strategy rifampicin-linezolid	Isoniazid (Week 16)	Isoniazid heteroresistance, alternating susceptible and resistant isolates on phenotypic testing detected at week 16 and relapse at week 48 (inhA mutation detected in resistant isolates on whole genome sequencing; absent in susceptible isolates); only one isolate at baseline (susceptible, no inhA mutation) prior to week 16 is insufficient to establish absence of heterogeneity at trial entry
TRUNCATE strategy rifampicin-linezolid	Pyrazinamide (Week 96)	Pyrazinamide resistance on phenotypic testing of the single isolate available at week 96 (no mutation detected on whole genome sequencing), no subsequent isolates available to confirm
TRUNCATE strategy rifampicin-linezolid	Pyrazinamide (Week 84)	Pyrazinamide resistance on phenotypic testing on isolates at week 84 and 86 (sequencing unsuccessful); pyrazinamide susceptible isolates on phenotypic testing at week 83 (no mutation detected on whole genome sequencing) and at week 88

Figure S1 Change in body weight from baseline to week 96, all arms

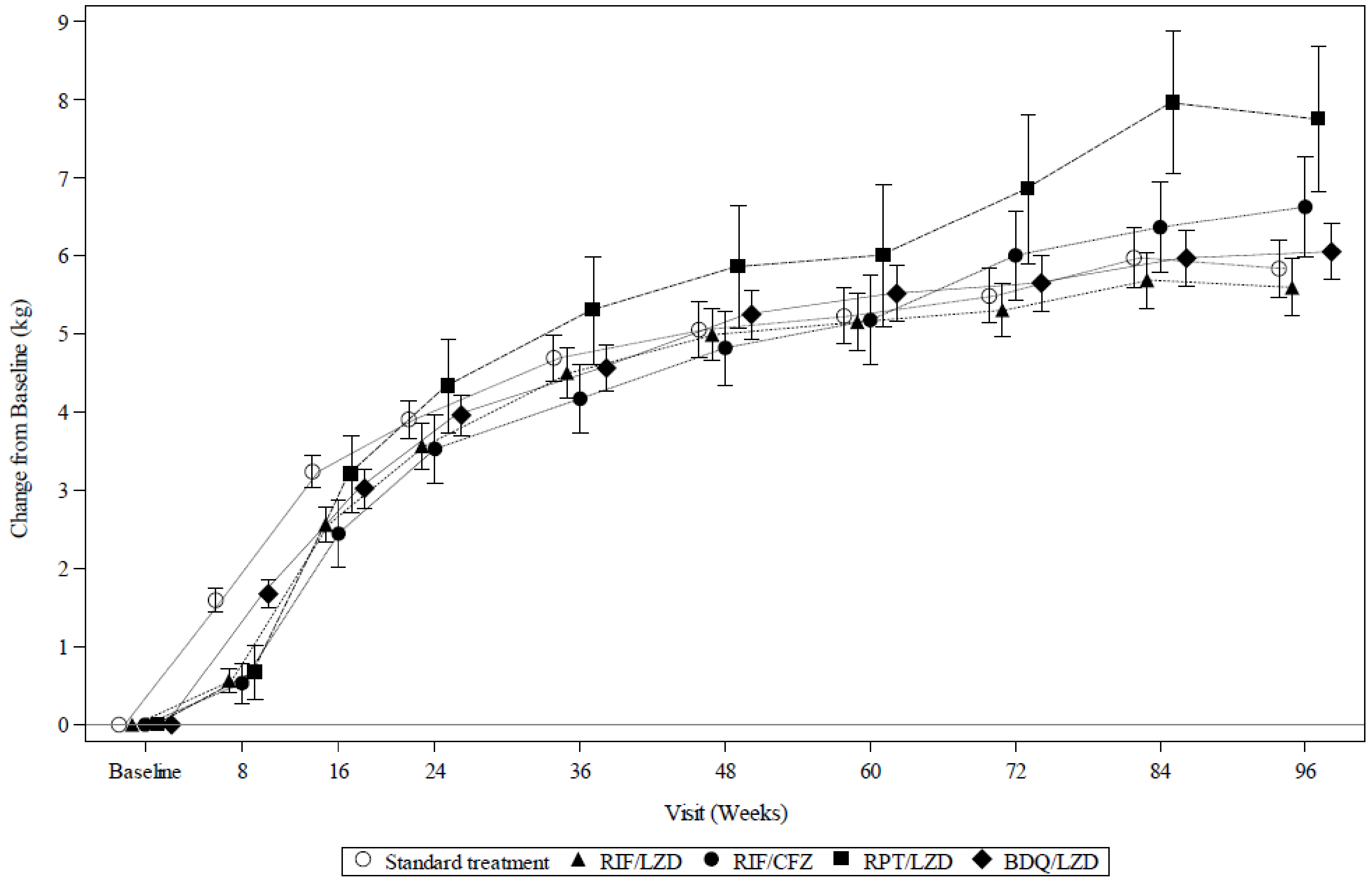

Figure shows the mean change in body weight from baseline in the standard treatment arm and each of the TRUNCATE strategy arms. Bars show the standard error of the mean.
